# Homogeneous production and characterization of recombinant *N*-GlcNAc-protein in *Pichia pastoris*

**DOI:** 10.1186/s12934-020-1280-0

**Published:** 2020-01-13

**Authors:** Shengjun Wang, Yongheng Rong, Yaoguang Wang, Decai Kong, Peng George Wang, Min Chen, Yun Kong

**Affiliations:** 1 National Glycoengineering Research Center and Shandong Key Laboratory of Carbohydrate Chemistry and Glycobiology, and State Key Laboratory of Microbial Technology, Shandong University, Qingdao, China; 2 School of Pharmaceutical Sciences, Sun Yat-sen University, Guangzhou, 510006 Guangdong China; 3 Department of General Surgery, Heze Municipal Hospital, Heze, 274000 Shandong China; 4 Department of Chemistry, Georgia State University, Atlanta, GA 30303 USA

**Keywords:** *Pichia pastoris*, Endoglycosidase, *N*-GlcNAc-protein, Glycoprotein, Homogeneous protein production

## Abstract

**Background:**

Therapeutic glycoproteins have occupied an extremely important position in the market of biopharmaceuticals. *N*-Glycosylation of protein drugs facilitates them to maintain optimal conformations and affect their structural stabilities, serum half-lives and biological efficiencies. Thus homogeneous *N*-glycoproteins with defined *N*-glycans are essential in their application in clinic therapeutics. However, there still remain several obstacles to acquire homogeneous *N*-glycans, such as the high production costs induced by the universal utilization of mammalian cell expression systems, the non-humanized *N*-glycan structures and the *N*-glycosylation microheterogeneities between batches.

**Results:**

In this study, we constructed a *Pichia pastoris* (*Komagataella phaffii*) *e*xpression system producing truncated *N*-GlcNAc-modified recombinant proteins through introducing an ENGase isoform (Endo-T) which possesses powerful hydrolytic activities towards high-mannose type *N*-glycans. The results showed that the location of Endo-T in different subcellular fractions, such as Endoplasmic reticulum (ER), Golgi or cell membrane, affected their hydrolytic efficiencies. When the Endo-T was expressed in Golgi, the secreted IgG1-Fc region was efficiently produced with almost completely truncated *N*-glycans and the *N*-GlcNAc modification on the glycosite Asn^297^ was confirmed via Mass Spectrometry.

**Conclusion:**

This strategy develops a simple glycoengineered yeast expression system to produce *N*-GlcNAc modified proteins, which could be further extended to different *N*-glycan structures. This system would provide a prospective platform for mass production of increasing novel glycoprotein drugs.

## Background

*N*-Linked glycosylation is a fundamental co- and/or posttranslational modification, regulating glycoprotein folding and functions. *N*-Glycosylation is evolutionarily conserved in all domains of life, including all eukaryotes, some bacteria [1] and many archaea [2]. In mammalian cells, most of the membrane-bound and secreted proteins are generally *N*-glycosylated and involved in many essential biological processes [3, 4]. In the classical pathway of *N*-linked glycosylation, the assembled oligosaccharide (GlcNAc_2_Man_9_Glc_3_) is transferred onto the asparagine (Asn) residue in the NXS/T (X ≠ Pro) context of the polypeptides from dolichol pyrophosphate by the oligosaccharyltransferases (OST) in endoplasmic reticulum [5–7] and glycans are subsequently maturated in the Golgi compartment [8].

At present, therapeutic glycoproteins have occupied an increasing proportion in the market of biopharmaceuticals. Glycoprotein drugs have been widely used to fight against diverse diseases, such as pathogenic microbial invasive diseases, autoimmune disorders and cancers. It has been shown that *N*-glycosylation and *N*-glycan structures can affect the biophysical and pharmacokinetic properties of therapeutic glycoproteins [9–11]. Several novel approaches have been attempted to engineer *N*-glycosylation pathway to decrease the microheterogeneity of therapeutic proteins via in vitro chemoenzymatic methods or in vivo engineered expression systems [11–18].

The endo-*N*-acetyl-β-D-glucosaminidase (endoglycosidase or ENGase) specifically cleave the diacetylchitobiose core [GlcNAc β (1–4) GlcNAc] of *N*-linked glycans between the two *N*-acetylglucosamine (GlcNAc) residues [19] to release an *N*-GlcNAc-carrying peptides/proteins and an intact oligosaccharide group [20]. Some ENGases or mutants also have potent transglycosylation activity [21–26] and were utilized in *N*-glycoprotein remodeling [27]. Wang and collaborators used an Endo-A mutant (N171A) to glycosylate IgG1-Fc region [21, 23, 28, 29], and further used the mutants of Endo-S (D233A and D233Q) or Endo-S2 (D184M and D184Q) for full-length antibody glycosylation remodeling with three major types (complex, high-mannose, and hybrid type) of *N*-glycans for modulating IgG effector function [14, 22, 30]. This chemoenzymatic glycosylation method utilizing ENGases provides an efficient way to introduce complex *N*-glycans onto polypeptides, which was valuable for glycoprotein drug production [13, 31]. In this method, *N*-GlcNAc modified proteins were essential as acceptors for the production of glycoproteins with different glycans. However, the direct transfer of a single GlcNAc moiety has only been found in the modification of specific serines or threonines catalyzed by *O*-linked GlcNAc transferase (OGT) [32]. Recently, *N*-Glycosyltransferase AaNGT and ApNGT^Q469A^ were reported to transfer GlcN and produce *N*-GlcNAc glycans by coupling with GlmA [16, 33].

*Pichia pastoris*, which was reassigned to the genus *Komagataella* spp. in 1995 [34], is an organism commonly employed to produce a variety of active proteins [35–37] with *N*- and/or *O*-linked glycans [38–40]. The *N*-linked glycans of the *P. pastoris*-produced proteins was high-mannose type without core fucose [41], which leads to reduced in vivo half-life and therapeutic function. The engineered *P. pastoris* have been constructed to produce glycoproteins with *N*-glycosylation profiles similar to human [39, 42], but the products are still heterogeneous with lower yield [39, 40, 43].

In this study, we construct a *P. pastoris* system expressing truncated *N*-GlcNAc-modified recombinant proteins through introducing an ENGase isoform (Endo-T) which possesses powerful hydrolytic activities towards high-mannose type *N*-glycan in intracellular environment, into different subcellular fractions. We believe the application of this easy and low-cost glycoprotein synthetic method would provide a prospective platform for mass production of increasing novel glycoprotein drugs with diverse homogeneous *N*-glycan structures.

## Results

### Expression of Endo-T on the surface of *Pichia pastoris*

**Fig. 1:**
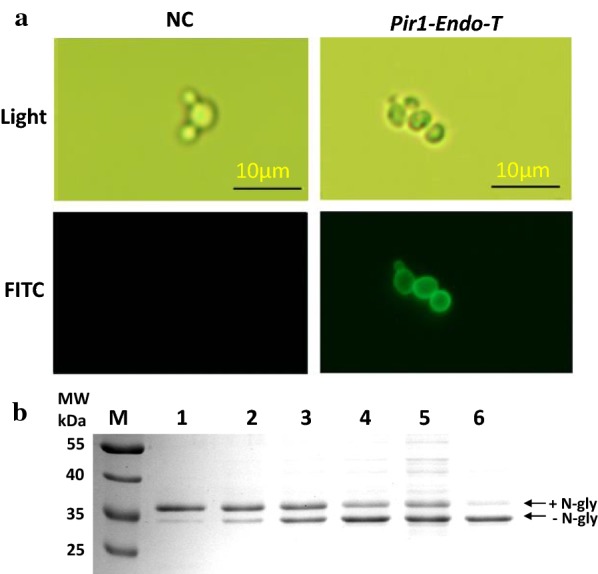
Endo-T expressed on the surface of *P. pastoris*. **a** Fluorescence micrographs showed the immunofluorescence staining of the *Pichia pastoris* WT (NC, left) and *Pir*-*Endo*-*T* (Right) with anti-Flag antibody. **b** SDS-PAGE was used to detect the deglycosylation activity of *P. pastoris Pir*-*Endo*-*T* strain. IgG1-Fc purified from *P. pastoris* GS115 was used as substrates to incubate at 37 °C for different time. Lane 1: 0 min; Lane 2: 1 h; Lane 3: 2 h; Lane 4: 4 h; Lane 5: 6 h; Lane 6: treated with PNGase F 1 h

Endo-T is the first fungal member of glycoside hydrolase family 18 with ENGase-type activity secreted from *Hypocrea jecorina* (*Trichoderma reesei*) [44]. In the GlycoDelete glycoengineering strategy, Endo-T has been successfully expressed in the Golgi of mammalian cells and plants to produce recombinant protein with homogenous *N*-glycan structures [17, 18], or to enhance integral membrane protein with homogenous *N*-GlcNAc expression in *P. pastoris* [45]. Here, we first expressed Endo-T on the surface of *P. pastoris* using the Pir1-based surface display system [46]. To detect the surface expression of Endo-T, immunofluorescence staining with anti-Flag antibody was performed. *P. pastoris* cells anchored with Endo-T were clearly labeled, while no immunofluorescence was observed in the cells transferred with an empty plasmid (Fig. 1a). This result indicated that the Endo-T could be successfully expressed on the cell surface. Human IgG1-Fc region and GalNAc-T1 recombinantly expressed in *P. pastoris* and Ribonuclease B (RNase B, Sigma) were used as the substrates to detect the deglycosylation activity of the immobilized Endo-T. Endo-T on the cell surface exhibited hydrolysis activity to remove high mannose-type *N*-glycans from different glycoproteins (Fig. 1b, Additional file 1: Figure S1). Compared with the commercial PNGase F, the surface displayed Endo-T showed lower deglycosylation efficiency (Fig. 1b, Additional file 1: Figure S1). PNGase F could release most of the glycans from IgG Fc domain in 1 h, while approximate 40% of the glycoprotein left after treatment with surface displayed Endo-T. We also tried to co-express human IgG1-Fc region in *P. pastoris* with surface displayed Endo-T and found most of the proteins still maintained the *N*-glycans (data not shown).

### Expression of ENGase in the ER or Golgi of *Pichia pastoris*

**Fig. 2:**
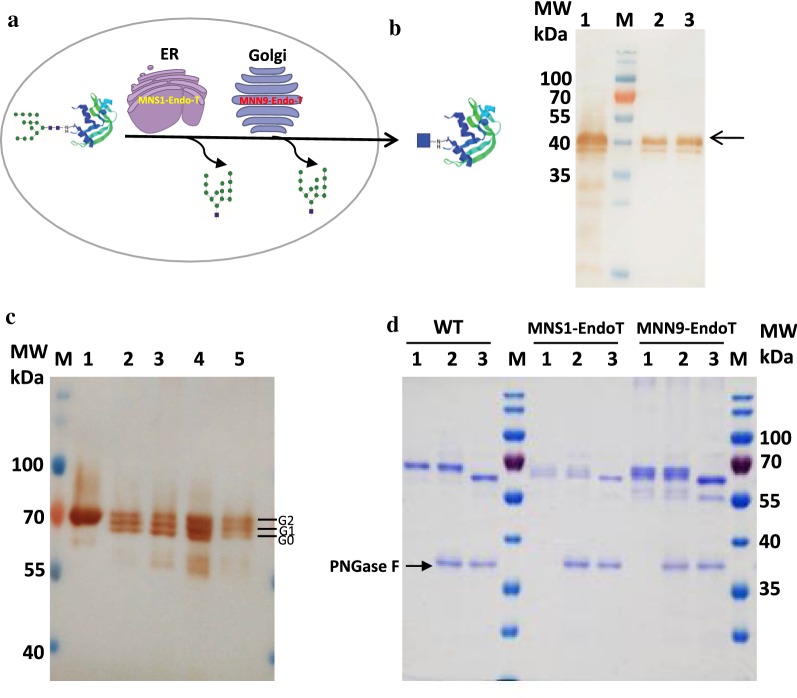
Endo-T expressed in Golgi or ER of *P. pastoris* to produce *N*-GlcNAc modified proteins. **a** Schematic presentation of the glycoengineering process of *P. pastoris* to produce *N*-GlcNAc modified proteins. **b** *P. pastoris* strains were detected using Western Blot with anti-Flag antibody. Lane 1: *P. pastoris Pir*-*Endo*-*T*; Lane 2: *P. pastoris MNS1*-*EndoT*; Lane 3: *P. pastoris MNN9*-*EndoT*; **c** human GalNAc-T1 secreted in different *P. pastoris* strains and detected using Western Blot with anti-His antibody. Lane 1: *P. pastoris* WT; Lanes 2–3: *P. pastoris MNS1*-*EndoT*; Lanes 4–5: *P. pastoris MNN9*-*EndoT.* G0–2 stands for the protein with 0–2 glycans. **d** Purified human GalNAc-T1 treated with PNGase F and analyzed by SDS-PAGE. Lane 1: before PNGase F treatment; Lane 2: treated with inactivated (boiled) PNGase F; Lane 3: treated with PNGase F. M stands for the protein marker

Endo-T has been expressed in the Golgi to produce recombinant protein with homogenous *N*-glycan structures [17]. Here, we first fused Endo-T with the trans-membrane region of *S. cerevisiae* MNN9 (mannosyltransferase) [47] or MNS1 (endoplasmic reticulum mannosyl-oligosaccharide 1,2-alpha-mannosidase) [48, 49] respectively, to ensure that Endo-T could be localized to the Golgi or Endoplasmic reticulum (ER). The fused proteins were expressed in *P. pastoris* to make a platform for the production of homogeneous *N*-GlcNAc modified proteins instead of heterogeneous high-mannose type *N*-glycans (Fig. 2a, b). In this study, human polypeptide *N*-acetylgalactosaminyltransferase 1 (GalNAc-T1) containing two *N*-glycans was selected to characterize the engineered yeast strains. The reporter protein construct built on the plasmid pPIC9K (Invitrogen) included the *Saccharomyces cerevisiae* α-mating factor signal at *N*-terminus to direct the protein to the ER membrane and a hexa-histidine tag at the C-terminus. Upon expression of the human GalNAc-T1 in the GS115 background, it was clear that the protein demonstrated only one protein band of approximately 70 kDa (Fig. 2c). By transferring to the engineered host strain, which expressed ENGases (Endo-T) in the ER or Golgi, the target proteins were produced with a similar yield, but exhibited three protein bands as shown in the SDS-PAGE and Western blot results (Fig. 2c). After in vitro treatment with PNGase F, all the samples showed a single band with similar MW (Fig. 2d), providing evidence that the lower bands in the samples from the engineered strains were the proteins deglycosylated of one or two *N*-glycans by Endo-T, although the deglycosylation efficiency is not high enough to remove all the *N*-glycans. Different fermentation conditions, such as the pH of culture medium (BMMY), methanol concentration and incubation temperature, were tested for the production of total and deglycosylated GalNAc-T1 (Additional file 1: Figures S2, S3, S4). The culture temperature showed great influence on the stability of GalNAc-T1 protein and low temperature (20 °C) was preferred. More deglycosylated GalNAc-T1 proteins was produced in *P. pastoris MNN9*-*EndoT* strains cultured in BMMY (with pH 6.0) for 4–5 days at 20 °C with 0.5% methanol (v/v) added to the culture every 24 h.

### Characterization of IgG1-Fc region with *N*-GlcNAc

**Fig. 3:**
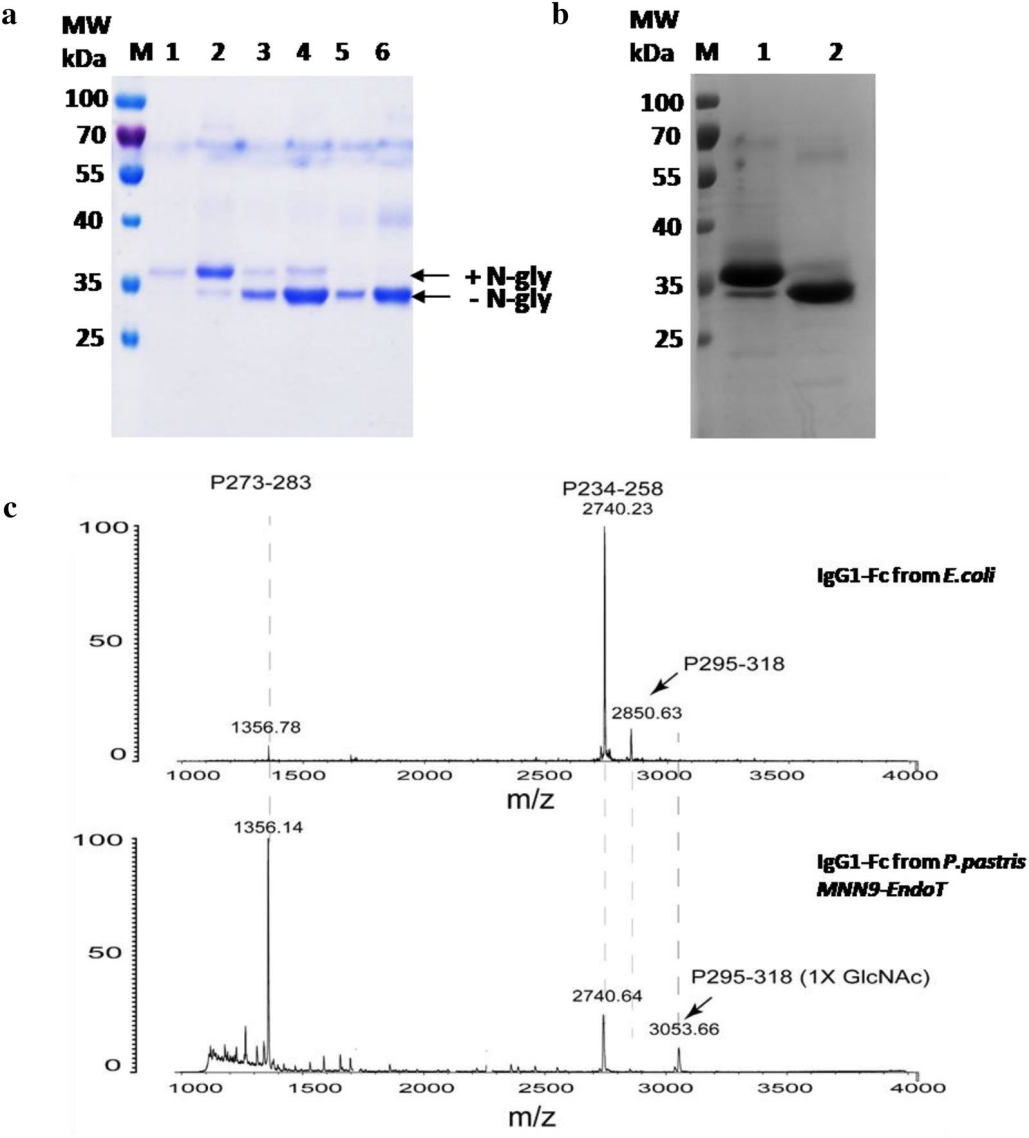
IgG1-Fc produced as an *N*-GlcNAc modified glycoform. **a** Human IgG1-Fc expressed in *P. pastoris* strains and detected with Coomassie staining SDS-PAGE. Lanes 1–2: *P. pastoris* WT cultured for 3 days and 4 days; Lanes 3–4: *P. pastoris MNS1*-*EndoT* cultured for 3 days and 4 days; Lanes 5–6: *P. pastoris MNN9*-*EndoT* cultured for 3 days and 4 days; M stands for the protein marker. **b** IgG1-Fc purified from *P. pastoris* WT (Lane 1) and *P. pastoris MNN9*-*EndoT* (Lane 2). M stands for the protein marker. **c** MALDI-TOF MS analysis of peptide maps from digested recombinant IgG1-Fc proteins. The IgG1-Fc proteins secreted from *E. coli* (upper) and *P. pastoris MNN9*-*EndoT* (lower) were digested with Glu-C, and analyzed by MALDI-TOF MS. The peak with m/z at 2850.63 was assigned as the peptide (P295-QYNSTYRVVSVLTVLHQDWLNGKE-318), while the peak with m/z at 3053.66 was assigned as the peptide (P295–318) with a HexNAc moiety addition

The IgG1-Fc region harboring an *N*-glycan moiety at Asn-297 [50] was selected to be expressed in the engineered strains. The full-length of human IgG1-Fc including the hinge region was cloned into the pPIC9k vector (Invitrogen) and the resulting recombinant plasmid was transformed into the engineered *P. pastoris* expression strain. After 4 or 5 day induction with 0.5% methanol, the supernatant of the medium were precipitated with acetone and detected by SDS-PAGE. The IgG1-Fc produced from *P. pastoris* wild type appeared as a protein band at ~ 38 kDa (Fig. 3a), which was in agreement with the calculated heterogeneous glycosylated monomeric IgG1-Fc (33–34 kDa). But when we expressed IgG1-Fc in the engineered yeast strains, the IgG1-Fc appeared a slightly smaller molecular weight (Fig. 3a). Thus, we estimated that the IgG1-Fc region expressed in the Endo-T-harboring strains could be deglycosylated. Moreover, more than 95% of the IgG1-Fc in *P. pastoris MNN9*-*EndoT* strains was deglycosylated, while approximate 10% of the IgG1-Fc in *P. pastoris MNS1*-*EndoT* was attached with *N*-glycans (Fig. 3a). The recombinant protein harvested from the *P. pastoris MNN9*-*EndoT* strains was then purified by affinity chromatography on a protein G column and approximate 200–250 mg of recombinant IgG1-Fc were obtained from 1 L of fermentation medium (Fig. 3b, Additional file 1: Figure S5), which was higher than the previous reports (from 10 to 100 mg/L) [51–53]. The purified IgG1-Fc from WT and *MNN9*-*EndoT* strain were detected by ConA blot (Additional file 1: Figure S6), suggesting the truncated *N*-glycan in engineered strain. To define whether the *N*-glycan structure was a single GlcNAc moiety, IgG1-Fc region proteins produced from *E. coli* and *P. pastoris MNN9*-*EndoT* strain were digested with Endoproteinase Glu-C and analyzed with MALDI-TOF MS (Fig. 3c) and LCMS-IT-TOF (Additional file 1: Figure S7). The protein from *P. pastoris* WT with the huge heterogeneous *N*-glycans was not easy to detect and compare with the protein from engineered strain (*MNN9*-*EndoT*) with one GlcNAc moiety. For IgG1-Fc from *E. coli*, a peak with the m/z value of 2850.63 was consistent with the expected naked peptide P295–318 (calculated, MW = 2850.183) (Fig. 3c, Additional file 2: Table S2). On the other hand, *N*-GlcNAc-IgG1-Fc from *P. pastoris MNN9*-*EndoT* strain assigned 3053.68 (m/z), indicating a HexNAc (an MW increase of 203 Da) addition in this peptide (Fig. 3c).

### Structural conformation of *N*-GlcNAc IgG1-Fc

**Fig. 4:**
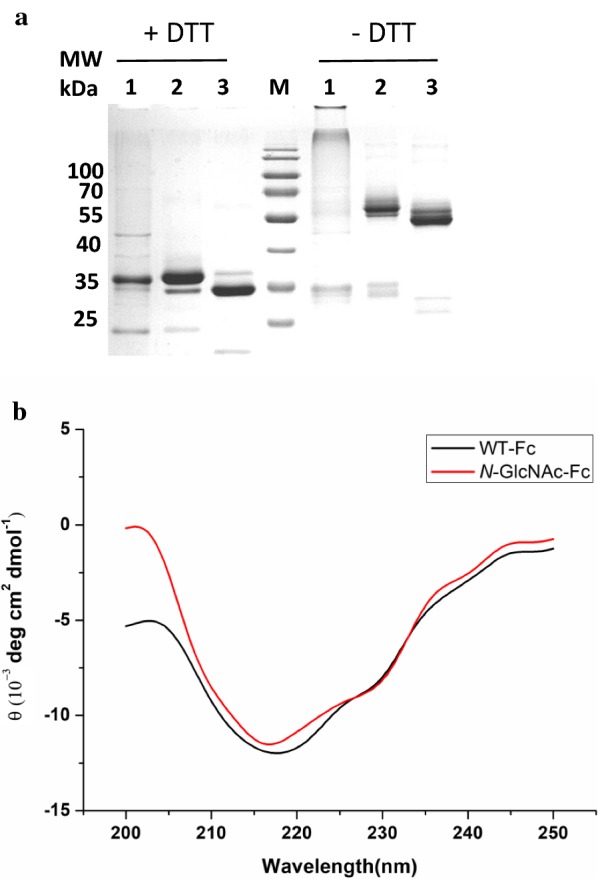
Characterization of the yeast expressed IgG1-Fc. **a** The purified IgG1-Fc proteins were detected with SDS-PAGE under non-reducing conditions (right) and reducing conditions (left). M stands for the protein marker; Lane 1: IgG1-Fc from *E. coli*; Lane 2: IgG1-Fc from *P. pastoris* WT; Lane 3: IgG1-Fc from *P. pastoris MNN9*-*EndoT*. **b** Comparative secondary structure content obtained by CD data analysis

The hinge-containing IgG1-Fc region should be covalently linked as a homodimer through the formation of a disulfide-bond [54]. SDS-PAGE with or without reduction was used to assay the forming of the dimer. On SDS-PAGE gel, the IgG1-Fc appeared as a protein band at ~ 38 kDa (from the WT strain) or ~ 34 kDa (from the engineered strain) under reducing conditions (with DTT treatment), while ~ 60 kDa (from the WT strain) or ~ 55-kDa (from the engineered strain) under non-reducing conditions (without DTT treatment) (Fig. 4a). The results were consistent with the previous observations [28]. We also found that the dimer appeared smaller in size on SDS-PAGE than the calculated molecular weight [28]. These results indicate that both *P. pastoris* recombinant IgG1-Fc proteins with or without the *N*-glycans were obtained as homodimers.

The secondary structures of IgG1-Fc regions expressed in *P. pastoris* were determined using far-UV circular dichroism (CD) spectroscopy (Fig. 4b). The IgG1-Fc region purified from *P. pastoris* WT strain and engineered *P. pastoris* were tested and compared. The secondary structure of the Fc fragment at 25 °C is populated primarily of beta-strands and a wavelength of 218 nm was chosen for unfolding by CD measurement [53]. For the WT-Fc, the spectra obtained at 25 °C showed a maximum negative peak at 218 nm, which was similar with previous reports [53]. Moreover, the CD spectrum of *N*-GlcNAc-Fc showed only minor differences to the WT spectrum (Fig. 4b), which was consistent with deglycosylated IgG [55] or aglycosylated Fc [56]. It can be seen that the Fc fragments with truncated glycans have intact secondary and tertiary structures that are very similar to the wild-type Fc fragment, with a characteristic minimum at 218 nm.

## Discussion

Glycoproteins are an important class of biomolecules involved in many physiological and pathological processes. Several strategies have been developed to produce glycoproteins with homogeneous glycan structures [11–14], of which ENGase-mediated *N*-glycan remodeling was a powerful approach to prepare defined glycoconjugates. The major limitation of this method is the difficulty to obtain *N*-GlcNAc proteins in large quantities. In this study, we constructed a *P. pastoris* expression system, which localized recombinant ENGases in the cell membrane, ER or Golgi, to produce secreted *N*-GlcNAc-modified proteins. Our results showed the location of ENGase in different subcellular fractions affected their hydrolytic efficiencies.

*Pichia pastoris* is an expression strain widely utilized to produce functional *N*-glycoproteins [35–37] with high yields [57]. The expression levels of recombinant proteins in *P. pastoris* were even up to 10 g/L [58]. The *N*-linked glycans from *P. pastoris* are of high mannose type without core fucose, which could be preferred as substrates by a variety of ENGase isoforms. We attempt to build up an expression system, which localized the recombinant ENGases in the cell surface membrane, ER or Golgi. As an immobilized enzyme on cell surface, the ENGase could hydrolyze glycans from *N*-glycoproteins in in vitro reaction system, while few deglycosylated proteins were found in the cultured medium containing methanol. When the ENGase was expressed in Golgi or ER, the secreted target glycoprotein could be efficiently deglycosylated. Fused with *MNN9*, the hydrolysis activity of ENGase against IgG Fc domain and GalNAc-T1 proteins is higher than fused with *MNS1*. It is assumed that the Endo-T preferred the microenvironment of yeast Golgi, such as the intracellular pH, as well as the glycan structure.

Human IgG1 carries a conserved *N*-glycan at Asn-297 of its Fc region. The presence and precise structures of this *N*-glycan plays an important role in determining antibody’s structure and effector functions. For example, the deglycosylated IgG1 are highly flexible and more prone to aggregation [59, 60]; removal of the core fucose from *N*-glycans increases the Fc’s affinity towards FcγRIIIA [14, 61–63]; the terminal α2, 6-sialylation is critical for its anti-inflammatory activity [64–66]. Fc region-containing fusion proteins are also influenced by the structure of *N*-glycans [67–69]. Both full length of human IgG1 and the IgG1-Fc region have been expressed in *P. pastoris* for glycan remodeling, in which the *N*-glycans need removing by in vitro reactions [14, 28]. When IgG1-Fc was expressed in our engineered strain (*MNN9*-*EndoT*), > 95% of secreted IgG1-Fc harbored only one GlcNAc moiety. Our results also showed that the total yield, the secondary structure and the protein conformation were not affected by the removal of the *N*-glycans. As the secreted proteins have been folded to the native state in the ER apparatus, the deglycosylation in the Golgi should only slightly affect the secretion of glycoproteins. Thus, *N*-GlcNAc IgG1-Fc protein produced from engineered *P. pastoris* should have the same properties as the in vitro deglycosylated proteins used for further *N*-glycans remodeling [14, 27, 30]. In our strategy, the *N*-GlcNAc proteins could be obtained with high yield via simple purification step from the culture medium.

Combined with the in vitro glycan remodeling or enzymatical elongation methods, this engineered *P. pastoris* system provides a prospective platform for powerful production of recombinant glycoprotein drugs. On the other hands, this system was not efficient enough to remove all the *N*-glycans when more than one oligosaccharide was attached on the target proteins. Some reasons might be responsible for the decrease of ENGase hydrolysis activity, such as (1) the spatial hindrance caused by localization expression; (2) the intracellular pH in Golgi was a non-optimal pH for Endo-T; (3) the cultured temperature (20–25 °C) was too low. But, the lower pH (pH 6.0) of the medium and the lower cultured temperature (20–25 °C) were important for higher yields of secreted recombinant proteins. The precise optimum pH of ENGases generally corresponds with the catalytic carboxylic acid residues in the enzyme active sites [70–72], and depends on the individual ENGase isoform [27]. The hydrolytic activity of ENGase was pH-dependent and drops rapidly as the pH is either higher or lower than the optimum pH [70]. The temperature was another factor to affect ENGases’ hydrolytic activity. Most of the novel ENGase isoforms are derived from microbes. Thus the optimum temperature is 30–37 °C and the lower temperature would decrease the hydrolytic activity. We supposed the temperature was the major reason for the lower deglycosylation efficiency of the fungal ENGase (Endo-T) in *P. pastoris* than in mammalian cells or plant cells. In the further work, we would screen and apply some novel ENGase isoforms which possess powerful hydrolytic activities towards high-mannose type *N*-glycan in the cultured condition of *P. pastoris*, such as pH 6.0, 20–25 °C.

## Conclusions

In this work, we developed a simple glycoengineered yeast expression system to efficiently produce homogeneous *N*-GlcNAc modified glycoproteins which could be further elongated to different *N*-glycan structures. We believe the application of this easy and low-cost glycoprotein synthetic method would provide a prospective platform to efficiently produce a growing number of novel glycoprotein drugs.

## Materials and methods

### Bacterial strains, media and chemicals

*Pichia pastoris* GS115 (his4^−^), pGAPZa and pPIC9K used for the protein expression were obtained from Invitrogen (Thermo Fisher Scientific). *Escherichia coli* TOP10 or DH5α strain was used as the host for recombinant DNA construction work. *E. coli* was grown in Luria–Bertani (LB) medium at 37 °C with 100 μg/mL ampicillin or 50 μg/mL zeocin where necessary. Buffered minimal glycerol (BMGY) medium, buffered minimal methanol (BMMY) medium and minimal dextrose (MD) medium were prepared following the *P. pastoris* expression manual (Invitrogen). Mouse anti-His monoclonal antibody and mouse anti-Flag monoclonal antibody were purchased from Genscript Bio-Technologies (Nanjing, China). Con A-Biotin was purchased from Vector Laboratories. HRP-conjugated secondary antibody and HRP- conjugated Streptavidin was purchased from ZSGB-Bio (Beijing, China). All other chemicals and solvents were bought from Sangon-Biotech (Shanghai, China).

### Plasmid construction and transformation

The genes (sequence in Additional file 2: Table S1) and primers (Table 1) used in this study were synthesized by Genscript Bio-Technologies. PCR was performed using relevant pairs of primers listed (Table 1). The *EndoT* gene was cloned into pPIC9K-*Pir1* with EcoRI and MluI to make the constructs pPIC9K-*Pir1*-*EndoT* and introduced into *P. pastoris* GS115 as previously reported [46]. The DNA encoding the transmembrane region of *S. cerevisiae* MNN9 (mannosyltransferase) or MNS1 (endoplasmic reticulum mannosyl-oligosaccharide 1,2-alpha-mannosidase) was fused with *EndoT* gene and cloned into pGAPZa with EcoRI and NotI to make the constructs pGAPZa-*MNN*9-*EndoT* or pGAPZa-*MNS1*-*EndoT* respectively. The plasmids were linearized with BspHI and introduced into *P. pastoris* GS115 via the Gene Pulser Xcell Electroporation System (Bio-Rad). The multicopy insert transformants were selected with YPD plates containing 1 mg/mL Zeocin. The Zeocin-resistant clones were confirmed by PCR with pGAP-F and EndoT-R.

**Table 1:** The primers used in this study

Primers	Sequence
MNS1-F	5-CCGGAAGGCGCCACCATGAAGAACTCTGTCGGTATTTCAATTGCAACCATTGTTGCTATCATAGCAG-3
MNS1-R	5-CCGCTCGAGTCTCTCAAAGTGTTCGTACCATGGCACATAGTATATAGCTGCTATGATAGCAACAATG-3
EndoT-F (EcoRI)	5-CGGAATTCGTTCCTGTCAAGGAGTTGCA-3
EndoT-R-Pir (MluI)	5-CGACGCGTTTACTTATCGTCATCGTCCT-3
EndoT-R (NotI)	5-ATAAGAATGCGGCCGCTTACTTATCGTCATCGTCCT-3
GalNAc-T1-F (SnaBI)	5-GACCTACGTAGGACTTCCTGCTGAAGATGT-3
GalNAc-T1-R (NotI)	5-ATAAGAATGCGGCCGCTAGTGATGATGATGATGATGATGGAATATTTCTGGCAGGGTGAC-3
Fc-F (EcoRI)	5-CCGGAATTCGAACCCAAGTCCTGCGAC-3
Fc-R (NotI)	5-ATAAGAATGCGGCCGCTCACTTGCCGGGGCTCAG-3
pGAP-F	5-GTCCCTATTTCAATCAATTGAA-3
5′AOX	5-GACTGGTTCCAATTGACAAGC-3

The cDNA encoding the human GalNAc-T1 and IgG1-Fc region were subcloned into the pPIC9K vector respectively. Resultant clones, named pPIC9k-*GALNT1* and pPIC9K-Fc, were selected and confirmed by DNA sequencing. The plasmid pPIC9k-*GALNT1* and pPIC9K-Fc were linearized with *Sac*I and introduced into *P. pastoris* GS115 WT and obtained pGAPZa-*MNN*9-*EndoT* and pGAPZa-*MNS1*-*EndoT* strains. The multicopy insert of transformants were selected with MD plates and subsequently YPD plates containing different concentrations of G418 (0.5 mg/mL, 1 mg/mL, 2 mg/mL or 4 mg/mL). The G418-resistant clones were confirmed by PCR with GalNAc-T1-F or Fc-F and 3′-*AOXI* primers. The PCR-positive clones from 4 mg/mL G418 plates were selected for the expression. Besides, the pET28a-IgG1-Fc was transferred into *E. coli* BL21 (DE3) as a control.

#### Analysis of engineered *P. pastoris* strains

The engineered *P. pastoris Pir1*-*EndoT* strains were cultured in BMMY medium with 0.5% methanol (v/v) for 12 h and washed with PBS. For immunofluorescence staining, the *P. pastoris* WT and *Pir*-*EndoT* strains were incubated with anti-Flag antibody and subsequently FITC-conjugated rabbit antibody against mouse Ig for 45 min and mounted with antifade reagent (BBI Life Sciences). Fluorescence microscopy was performed using a Zeiss Axioskop 2 plus with an AxioCam MR3. Bit depth and pixel dimensions were 36 bits and 1388 × 1040 pixels, respectively. For western blot, the *P. pastoris* strains were lysed with glass beads and analyzed by Western blot with anti-Flag antibody.

#### Expression and purification of recombinant proteins

Recombinant yeast clones were grown at 30 °C in 50 mL BMGY until the OD_600_ reached 2–6. For the fermentation condition screen, Cells were harvested and cultured in BMMY (with pH 6.0, 6.5 or 7.0) for 4–5 days at different temperature (20 °C or 25 °C) and 0.5% or 1% methanol (v/v) was added to the culture every 24 h. The fermentation culture was precipitated by cold acetone after 2–5 days respectively and Coomassie-stained SDS-PAGE was used to test the production of total and glycosylated proteins.

After fermentation, secreted recombinant proteins were purified using Ni–NTA agarose (for GlalNAc-T1) or Protein G column (for IgG1-Fc region). For GalNAc-T1, the cell-free supernatant was loaded onto the Ni–NTA column pre-equilibrated with binding buffer (20 mM Tris, pH 8.0, 150 mM NaCl, 20 mM imidazole). After washed with 30 mL of binding buffer, the purified proteins were eluted with binding buffer containing 250 mM imidazole. For IgG1-Fc region, the cell-free supernatant was diluted 5 times by PBS buffer, and was loaded onto the Protein G column pre-equilibrated with PBS buffer. After washed with 30 mL of PBS buffer, the purified proteins were eluted with 0.1 M Glycine Buffer pH 2.7. The eluted protein was neutralized immediately with 1 M Tris–HCl (pH 7.0). The positive fractions (determined by SDS-PAGE) were desalted and stored at − 20 °C. Recombinant IgG1-Fc region produced in *E. coli* was purified following the same Ni–NTA protocol.

#### SDS-PAGE and western blot

Purified IgG1-Fc region and GalNAc-T1 proteins were treated with peptide *N*-glycosidase F (PNGase F, New England Biolabs), following the manufacturer’s protocol. Samples were run on 12% SDS-PAGE gels with or without DTT reduction, and transferred onto polyvinylidene fluoride membranes for 90 min. After blocked in 5% BSA or 1% polyvinylpyrrolidone (Sigma) the membranes were incubated with His-tag antibody or ConA-B respectively at 4 °C overnight. Blots were developed with DAB Substrate kit (Solarbio, China) following incubation with HRP-conjugated secondary antibody for 1 h at room temperature.

#### Mass spectrometric analysis of IgG1-Fc protein

Approximately 20 μg of Fc protein was reduced with 10 mM DTT in 50 mM ammonium bicarbonate (AmBic) for 45 min at 60 °C and alkylated by 20 mM iodoacetamide at room temperate for 30 min. Then, 10 mM DTT was added to terminate alkylation before the protein was subjected to proteolysis by Glu-C (Promega). The treatment was terminated by boiling, and the digested peptides were desalted via a standard C18 Zip-Tip procedure and analyzed by MALDI-TOF MS (Shimadzu, Tokyo, Japan) or LCMS-IT-TOF system (Shimadzu, Tokyo, Japan) operated in the positive linear mode.

#### Circular dichroism spectroscopy

The secondary structure of the IgG1-Fc domian (from *P. pastoris* WT and *MNN9*-*EndoT* strains) were determined by circular dichroism using J-815 Jasco spectropolarimeter (Jasco Co., Tokyo, Japan) equipped with a PTC-348 WI thermostat under a constant nitrogen flow. A 0.1-cm path length cell was used to collect data in the far ultraviolet region (200–250 nm) at a scan speed of 20 nm/min and a response time of 1 s. Spectra were acquired at 25 °C and measured in PBS buffer. The spectrum of a blank containing buffer alone was subtracted from all spectra. The CD data were analyzed using the CDtoolX and online tools dichroweb (http://dichroweb.cryst.bbk.ac.uk/).

## Supplementary information

**Additional file 1: Figure S1.** SDS-PAGE was used to detect the deglycosylation activity of *P. pastoris Pir-Endo-T* strain. RNase B (a, *upper*) and GalNAc-T1 purified from *P. pastoris* GS115 (b, *lower*) were used as substrates to incubate at 37 °C for different time. Lane 1: 0 min; Lane 2: 1 h; Lane 3: 2 h; Lane 4: 4 h; Lane 5: 6 h; Lane 6:8 h; Lane 7: over-night; Lane 8: treated with PNGase F 1 h. The star showed the bands from *P. pastoris* strain. **Figure S2.** SDS-PAGE analysis of GalNAc-T1 expression in engineered strains at 20 °C. The *P. pastoris MNS1-EndoT* strain (a and b) and *P. pastoris MNN9-EndoT* (c and d) were cultured in BMMY with different pH at 20 °C, and 0.5% (a and c) or 1% (b and d) methanol (v/v) was added to the culture every 24 h. Lane 1: 2d; Lane 2: 3d; Lane 3: 4d; Lane 4: 5d. **Figure S3.** SDS-PAGE analysis of GalNAc-T1 expression in engineered strain at 25 °C. The *P. pastoris MNS1-EndoT* strain (a and b) and *P. pastoris MNN9-EndoT* (c and d) was cultured in BMMY with different pH at 25 °C, and 0.5% (a and c) or 1% (b and d) methanol (v/v) was added to the culture every 24 h. Lane 1: 2d; Lane 2: 3d; Lane 3: 4d; Lane 4: 5d. **Figure S4.** SDS-PAGE analysis of GalNAc-T1 expression in engineered strain. The *P. pastoris MNS1-EndoT* strain (Right) and *P. pastoris MNN9-EndoT* (Left) was cultured in BMMY with pH 6.0 at different temperature and different concentration of methanol (v/v) was added to the culture every 24 h. Lane 1: 20 °C 0.5% Methanol 2d; Lane 2: 20 °C 0.5% Methanol, 3d; Lane 3: 25 °C 0.5% Methanol, 2d; Lane 4: 25 °C 0.5% Methanol 3d; Lane 5: 30 °C 0.5% Methanol, 2d; Lane 6: 30 °C 0.5% Methanol, 3d; Lane 7: 20 °C 0.2% Methanol 2d; Lane 8: 20 °C 0.2% Methanol 3d; Lane 9: 20 °C 0.1% Methanol 2d; Lane 10: 20 °C 0.1% Methanol 3d. **Figure S5.** The purification of IgG1-Fc. IgG1-Fc from *E. coli* was purified with Ni-NTA and IgG1-Fc from *P. pastoris* was purified with Protein G column. The numbers showed the different eluted fractions. **Figure S6.** SDS-PAGE and lectin blot analysis of IgG Fc protein. IgG1-Fc purified from *P. pastoris* WT (Lane 1) and *P. pastoris MNN9-EndoT* (Lane 2) were analyzed with Coomassie blue (left) or Con A lectin blot (right). **Figure S7.** LC/MS-IT-TOF MS analysis of peptide maps from digested recombinant IgG1-Fc proteins. The IgG1-Fc protein from *E. coli* (upper) and *P. pastoris MNN9-EndoT* (lower) were digested with Glu-C, and analyzed by LC/MS-IT-TOF. The peak with m/z 713.6287 was assigned as the peptide (P295-QYNSTYRVVSVLTVLHQDWLNGKE-318), while the peak with m/z at 764.3955 was assigned as the peptide (P295–318) with a HexNAc moiety. (4) stands for [M+4H]^4+^.

**Additional file 2: Table S1.** The DNA and amino acid sequences used in this study. **Table S2.** Peptide map of recombinant IgG1-Fc domain digested with Glc-C.

## Data Availability

Not applicable.

## Abbreviations

ENGase: endo-beta-*N*-acetylglucosaminidase or endoglycosidase
Asn: asparagine
BMGY: buffered minimal glycerol medium
BMMY: buffered minimal methanol medium
ER: endoplasmic reticulum
GalNAc-T1: polypeptide *N*-acetylgalactosaminyltransferase 1
PBS: phosphate buffered saline
YPD: rich yeast medium

## References

[1] Nothaft H, Szymanski CM (2010). Protein glycosylation in bacteria: sweeter than ever. Nat Rev Microbiol.

[2] Calo D, Kaminski L, Eichler J (2010). Protein glycosylation in Archaea: sweet and extreme. Glycobiology.

[3] Dwek RA (1996). Glycobiology: toward understanding the function of sugars. Chem Rev.

[4] Marth JD, Grewal PK (2008). Mammalian glycosylation in immunity. Nat Rev Immunol.

[5] Dell A, Galadari A, Sastre F, Hitchen P (2010). Similarities and differences in the glycosylation mechanisms in prokaryotes and eukaryotes. Int J Microbiol.

[6] Abu-Qarn M, Eichler J, Sharon N (2008). Not just for Eukarya anymore: protein glycosylation in Bacteria and Archaea. Curr Opin Struct Biol.

[7] Burda P, Aebi M (1999). The dolichol pathway of *N*-linked glycosylation. Biochim Biophys Acta.

[8] Helenius A, Aebi M (2001). Intracellular functions of *N*-linked glycans. Science.

[9] Elliott S, Lorenzini T, Asher S, Aoki K, Brankow D, Buck L, Busse L, Chang D, Fuller J, Grant J (2003). Enhancement of therapeutic protein in vivo activities through glycoengineering. Nat Biotechnol.

[10] Stork R, Zettlitz KA, Muller D, Rether M, Hanisch FG, Kontermann RE (2008). *N*-Glycosylation as novel strategy to improve pharmacokinetic properties of bispecific single-chain diabodies. J Biol Chem.

[11] Lizak C, Fan YY, Weber TC, Aebi M (2011). *N*-Linked glycosylation of antibody fragments in *Escherichia coli*. Bioconjug Chem.

[12] Wacker M, Linton D, Hitchen PG, Nita-Lazar M, Haslam SM, North SJ, Panico M, Morris HR, Dell A, Wren BW, Aebi M (2002). *N*-Linked glycosylation in *Campylobacter jejuni* and its functional transfer into *E. coli*. Science.

[13] Lomino JV, Naegeli A, Orwenyo J, Amin MN, Aebi M, Wang LX (2013). A two-step enzymatic glycosylation of polypeptides with complex *N*-glycans. Bioorg Med Chem.

[14] Li T, DiLillo DJ, Bournazos S, Giddens JP, Ravetch JV, Wang LX (2017). Modulating IgG effector function by Fc glycan engineering. Proc Natl Acad Sci USA.

[15] Yang Z, Wang S, Halim A, Schulz MA, Frodin M, Rahman SH, Vester-Christensen MB, Behrens C, Kristensen C, Vakhrushev SY (2015). Engineered CHO cells for production of diverse, homogeneous glycoproteins. Nat Biotechnol.

[16] Kong Y, Li J, Hu X, Wang Y, Meng Q, Gu G, Wang PG, Chen M (2018). *N*-Glycosyltransferase from *Aggregatibacter aphrophilus* synthesizes glycopeptides with relaxed nucleotide-activated sugar donor selectivity. Carbohydr Res.

[17] Meuris L, Santens F, Elson G, Festjens N, Boone M, Dos Santos A, Devos S, Rousseau F, Plets E, Houthuys E (2014). GlycoDelete engineering of mammalian cells simplifies *N*-glycosylation of recombinant proteins. Nat Biotechnol.

[18] Piron R, Santens F, De Paepe A, Depicker A, Callewaert N (2015). Using GlycoDelete to produce proteins lacking plant-specific *N*-glycan modification in seeds. Nat Biotechnol.

[19] Maley F, Trimble RB, Tarentino AL, Plummer TH (1989). Characterization of glycoproteins and their associated oligosaccharides through the use of endoglycosidases. Anal Biochem.

[20] Stals I, Karkehabadi S, Kim S, Ward M, Van Landschoot A, Devreese B, Sandgren M (2012). High resolution crystal structure of the endo-*N*-acetyl-beta-d-glucosaminidase responsible for the deglycosylation of *Hypocrea jecorina* cellulases. PLoS ONE.

[21] Huang W, Yang Q, Umekawa M, Yamamoto K, Wang LX (2010). Arthrobacter endo-beta-*N*-acetylglucosaminidase shows transglycosylation activity on complex-type *N*-glycan oxazolines: one-pot conversion of ribonuclease B to sialylated ribonuclease C. ChemBioChem.

[22] Huang W, Giddens J, Fan SQ, Toonstra C, Wang LX (2012). Chemoenzymatic glycoengineering of intact IgG antibodies for gain of functions. J Am Chem Soc.

[23] Huang W, Li C, Li B, Umekawa M, Yamamoto K, Zhang X, Wang LX (2009). Glycosynthases enable a highly efficient chemoenzymatic synthesis of *N*-glycoproteins carrying intact natural *N*-glycans. J Am Chem Soc.

[24] Umekawa M, Huang W, Li B, Fujita K, Ashida H, Wang LX, Yamamoto K (2008). Mutants of *Mucor hiemalis* endo-beta-*N*-acetylglucosaminidase show enhanced transglycosylation and glycosynthase-like activities. J Biol Chem.

[25] Li B, Zeng Y, Hauser S, Song H, Wang LX (2005). Highly efficient endoglycosidase-catalyzed synthesis of glycopeptides using oligosaccharide oxazolines as donor substrates. J Am Chem Soc.

[26] Yin J, Li L, Shaw N, Li Y, Song JK, Zhang W, Xia C, Zhang R, Joachimiak A, Zhang HC (2009). Structural basis and catalytic mechanism for the dual functional endo-beta-*N*-acetylglucosaminidase A. PLoS ONE.

[27] Fairbanks AJ (2017). The ENGases: versatile biocatalysts for the production of homogeneous *N*-linked glycopeptides and glycoproteins. Chem Soc Rev.

[28] Wei Y, Li C, Huang W, Li B, Strome S, Wang LX (2008). Glycoengineering of human IgG1-Fc through combined yeast expression and in vitro chemoenzymatic glycosylation. Biochemistry.

[29] Heidecke CD, Ling Z, Bruce NC, Moir JW, Parsons TB, Fairbanks AJ (2008). Enhanced glycosylation with mutants of endohexosaminidase A (endo A). ChemBioChem.

[30] Li T, Tong X, Yang Q, Giddens JP, Wang LX (2016). Glycosynthase mutants of endoglycosidase S2 show potent transglycosylation activity and remarkably relaxed substrate specificity for antibody glycosylation remodeling. J Biol Chem.

[31] Li B, Song H, Hauser S, Wang LX (2006). A highly efficient chemoenzymatic approach toward glycoprotein synthesis. Org Lett.

[32] Hart GW, Housley MP, Slawson C (2007). Cycling of *O*-linked beta-*N*-acetylglucosamine on nucleocytoplasmic proteins. Nature.

[33] Xu Y, Wu Z, Zhang P, Zhu H, Song Q, Wang L, Wang F, Wang PG, Cheng J (2017). A novel enzymatic method for synthesis of glycopeptides carrying natural eukaryotic *N*-glycans. Chem Commun.

[34] Yamada Y, Matsuda M, Maeda K, Mikata K (1995). The phylogenetic relationships of methanol-assimilating yeasts based on the partial sequences of 18S and 26S ribosomal RNAs: the proposal of *Komagataella* gen. nov. (Saccharomycetaceae). Biosci Biotechnol Biochem.

[35] Cregg JM, Cereghino JL, Shi J, Higgins DR (2000). Recombinant protein expression in *Pichia pastoris*. Mol Biotechnol.

[36] Cregg JM, Vedvick TS, Raschke WC (1993). Recent advances in the expression of foreign genes in *Pichia pastoris*. Nat Biotechnol.

[37] Mochizuki S, Hamato N, Hirose M, Miyano K, Ohtani W, Kameyama S, Kuwae S, Tokuyama T, Ohi H (2001). Expression and characterization of recombinant human antithrombin III in *Pichia pastoris*. Protein Expr Purif.

[38] Kannan V, Narayanaswamy P, Gadamsetty D, Hazra P, Khedkar A, Iyer H (2009). A tandem mass spectrometric approach to the identification of *O*-glycosylated glargine glycoforms in active pharmaceutical ingredient expressed in *Pichia pastoris*. Rapid Commun Mass Spectrom.

[39] Hamilton SR, Davidson RC, Sethuraman N, Nett JH, Jiang Y, Rios S, Bobrowicz P, Stadheim TA, Li H, Choi BK (2006). Humanization of yeast to produce complex terminally sialylated glycoproteins. Science.

[40] Li H, Sethuraman N, Stadheim TA, Zha D, Prinz B, Ballew N, Bobrowicz P, Choi BK, Cook WJ, Cukan M (2006). Optimization of humanized IgGs in glycoengineered *Pichia pastoris*. Nat Biotechnol.

[41] Gemmill TR, Trimble RB (1999). Overview of *N*- and *O*-linked oligosaccharide structures found in various yeast species. Biochim Biophys Acta.

[42] Jacobs PP, Geysens S, Vervecken W, Contreras R, Callewaert N (2009). Engineering complex-type *N*-glycosylation in *Pichia pastoris* using GlycoSwitch technology. Nat Protoc.

[43] Beck A, Cochet O, Wurch T (2010). GlycoFi’s technology to control the glycosylation of recombinant therapeutic proteins. Expert Opin Drug Discov.

[44] Stals I, Samyn B, Sergeant K, White T, Hoorelbeke K, Coorevits A, Devreese B, Claeyssens M, Piens K (2010). Identification of a gene coding for a deglycosylating enzyme in *Hypocrea jecorina*. FEMS Microbiol Lett.

[45] Claes K, Vandewalle K, Laukens B, Laeremans T, Vosters O, Langer I, Parmentier M, Steyaert J, Callewaert N (2016). Modular integrated secretory system engineering in *Pichia pastoris* to enhance G-protein coupled receptor expression. ACS Synth Biol.

[46] Wang Q, Li L, Chen M, Qi Q, Wang PG (2008). Construction of a novel *Pichia pastoris* cell-surface display system based on the cell wall protein Pir1. Curr Microbiol.

[47] Jungmann J, Munro S (1998). Multi-protein complexes in the cis Golgi of *Saccharomyces cerevisiae* with alpha-1,6-mannosyltransferase activity. EMBO J.

[48] Puccia R, Grondin B, Herscovics A (1993). Disruption of the processing alpha-mannosidase gene does not prevent outer chain synthesis in *Saccharomyces cerevisiae*. Biochem J.

[49] Banerjee S, Vishwanath P, Cui J, Kelleher DJ, Gilmore R, Robbins PW, Samuelson J (2007). The evolution of *N*-glycan-dependent endoplasmic reticulum quality control factors for glycoprotein folding and degradation. Proc Natl Acad Sci USA.

[50] Zou G, Ochiai H, Huang W, Yang Q, Li C, Wang LX (2011). Chemoenzymatic synthesis and Fcgamma receptor binding of homogeneous glycoforms of antibody Fc domain. Presence of a bisecting sugar moiety enhances the affinity of Fc to FcgammaIIIa receptor. J Am Chem Soc.

[51] Alsenaidy MA, Okbazghi SZ, Kim JH, Joshi SB, Middaugh CR, Tolbert TJ, Volkin DB (2014). Physical stability comparisons of IgG1-Fc variants: effects of *N*-glycosylation site occupancy and Asp/Gln residues at site Asn 297. J Pharm Sci.

[52] Guo Y, Kang W, Zhong Y, Li R, Li G, Shen Y, Hu S, Sun J, Xiao W (2012). Purification and characterization of human IL-10/Fc fusion protein expressed in *Pichia pastoris*. Protein Expr Purif.

[53] Wozniak-Knopp G, Stadlmann J, Ruker F (2012). Stabilisation of the Fc fragment of human IgG1 by engineered intradomain disulfide bonds. PLoS ONE.

[54] Lund J, Takahashi N, Popplewell A, Goodall M, Pound JD, Tyler R, King DJ, Jefferis R (2000). Expression and characterization of truncated forms of humanized L243 IgG1. Architectural features can influence synthesis of its oligosaccharide chains and affect superoxide production triggered through human Fcgamma receptor I. Eur J Biochem.

[55] Wyss DF, Choi JS, Li J, Knoppers MH, Willis KJ, Arulanandam AR, Smolyar A, Reinherz EL, Wagner G (1995). Conformation and function of the *N*-linked glycan in the adhesion domain of human CD2. Science.

[56] Yageta S, Shibuya R, Imamura H, Honda S (2017). Conformational and colloidal stabilities of human immunoglobulin G Fc and its cyclized variant: independent and compensatory participation of domains in aggregation of multidomain proteins. Mol Pharm.

[57] Cereghino GP, Cereghino JL, Ilgen C, Cregg JM (2002). Production of recombinant proteins in fermenter cultures of the yeast *Pichia pastoris*. Curr Opin Biotechnol.

[58] Cereghino JL, Cregg JM (2000). Heterologous protein expression in the methylotrophic yeast *Pichia pastoris*. FEMS Microbiol Rev.

[59] Zheng K, Bantog C, Bayer R (2011). The impact of glycosylation on monoclonal antibody conformation and stability. MAbs.

[60] Krapp S, Mimura Y, Jefferis R, Huber R, Sondermann P (2003). Structural analysis of human IgG-Fc glycoforms reveals a correlation between glycosylation and structural integrity. J Mol Biol.

[61] Lin CW, Tsai MH, Li ST, Tsai TI, Chu KC, Liu YC, Lai MY, Wu CY, Tseng YC, Shivatare SS (2015). A common glycan structure on immunoglobulin G for enhancement of effector functions. Proc Natl Acad Sci USA.

[62] Umana P, Jean-Mairet J, Moudry R, Amstutz H, Bailey JE (1999). Engineered glycoforms of an antineuroblastoma IgG1 with optimized antibody-dependent cellular cytotoxic activity. Nat Biotechnol.

[63] Ferrara C, Grau S, Jager C, Sondermann P, Brunker P, Waldhauer I, Hennig M, Ruf A, Rufer AC, Stihle M (2011). Unique carbohydrate-carbohydrate interactions are required for high affinity binding between FcgammaRIII and antibodies lacking core fucose. Proc Natl Acad Sci USA.

[64] Kaneko Y, Nimmerjahn F, Ravetch JV (2006). Anti-inflammatory activity of immunoglobulin G resulting from Fc sialylation. Science.

[65] Anthony RM, Nimmerjahn F, Ashline DJ, Reinhold VN, Paulson JC, Ravetch JV (2008). Recapitulation of IVIG anti-inflammatory activity with a recombinant IgG Fc. Science.

[66] Anthony RM, Wermeling F, Karlsson MC, Ravetch JV (2008). Identification of a receptor required for the anti-inflammatory activity of IVIG. Proc Natl Acad Sci USA.

[67] Keck R, Nayak N, Lerner L, Raju S, Ma S, Schreitmueller T, Chamow S, Moorhouse K, Kotts C, Jones A (2008). Characterization of a complex glycoprotein whose variable metabolic clearance in humans is dependent on terminal *N*-acetylglucosamine content. Biologicals.

[68] Kogelberg H, Tolner B, Sharma SK, Lowdell MW, Qureshi U, Robson M, Hillyer T, Pedley RB, Vervecken W, Contreras R (2007). Clearance mechanism of a mannosylated antibody-enzyme fusion protein used in experimental cancer therapy. Glycobiology.

[69] Liu L, Gomathinayagam S, Hamuro L, Prueksaritanont T, Wang W, Stadheim TA, Hamilton SR (2013). The impact of glycosylation on the pharmacokinetics of a TNFR2: Fc fusion protein expressed in glycoengineered *Pichia pastoris*. Pharm Res.

[70] Schmidt BF, Ashizawa E, Jarnagin AS, Lynn S, Noto G, Woodhouse L, Estell DA, Lad P (1994). Identification of two aspartates and a glutamate essential for the activity of endo-beta-*N*-acetylglucosaminidase H from *Streptomyces plicatus*. Arch Biochem Biophys.

[71] Kadowaki S, Yamamoto K, Fujisaki M, Izumi K, Tochikura T, Yokoyama T (1990). Purification and characterization of a novel fungal endo-beta-*N*-acetylglucosaminidase acting on complex oligosaccharides of glycoproteins. Agric Biol Chem.

[72] Abbott DW, Macauley MS, Vocadlo DJ, Boraston AB (2009). Streptococcus pneumoniae endohexosaminidase D, structural and mechanistic insight into substrate-assisted catalysis in family 85 glycoside hydrolases. J Biol Chem.

