# Supplementary material for: Homogeneous production and characterization of recombinant N-GlcNAc-protein in Pichia pastoris
Source: Microb Cell Fact. 2020 Jan 13;19:7. doi: 10.1186/s12934-020-1280-0 (PMC6956495; doi:10.1186/s12934-020-1280-0)
Supplement: Supplementary file 1 — Additional file 1: Figure S1. SDS-PAGE was used to detect the deglycosylation activity of P. pastoris Pir-Endo-T strain. RNase B (a, upper) and GalNAc-T1 purified from P. pastoris GS115 (b, lower) were used as substrates to incubate at 37 °C for different time. Lane 1: 0 min; Lane 2: 1 h; Lane 3: 2 h; Lane 4: 4 h; Lane 5: 6 h; Lane 6:8 h; Lane 7: over-night; Lane 8: treated with PNGase F 1 h. The star showed the bands from P. pastoris strain. Figure S2. SDS-PAGE analysis of GalNAc-T1 expression in engineered strains at 20 °C. The P. pastoris MNS1-EndoT strain (a and b) and P. pastoris MNN9-EndoT (c and d) were cultured in BMMY with different pH at 20 °C, and 0.5% (a and c) or 1% (b and d) methanol (v/v) was added to the culture every 24 h. Lane 1: 2d; Lane 2: 3d; Lane 3: 4d; Lane 4: 5d. Figure S3. SDS-PAGE analysis of GalNAc-T1 expression in engineered strain at 25 °C. The P. pastoris MNS1-EndoT strain (a and b) and P. pastoris MNN9-EndoT (c and d) was cultured in BMMY with different pH at 25 °C, and 0.5% (a and c) or 1% (b and d) methanol (v/v) was added to the culture every 24 h. Lane 1: 2d; Lane 2: 3d; Lane 3: 4d; Lane 4: 5d. Figure S4. SDS-PAGE analysis of GalNAc-T1 expression in engineered strain. The P. pastoris MNS1-EndoT strain (Right) and P. pastoris MNN9-EndoT (Left) was cultured in BMMY with pH 6.0 at different temperature and different concentration of methanol (v/v) was added to the culture every 24 h. Lane 1: 20 °C 0.5% Methanol 2d; Lane 2: 20 °C 0.5% Methanol, 3d; Lane 3: 25 °C 0.5% Methanol, 2d; Lane 4: 25 °C 0.5% Methanol 3d; Lane 5: 30 °C 0.5% Methanol, 2d; Lane 6: 30 °C 0.5% Methanol, 3d; Lane 7: 20 °C 0.2% Methanol 2d; Lane 8: 20 °C 0.2% Methanol 3d; Lane 9: 20 °C 0.1% Methanol 2d; Lane 10: 20 °C 0.1% Methanol 3d. Figure S5. The purification of IgG1-Fc. IgG1-Fc from E. coli was purified with Ni-NTA and IgG1-Fc from P. pastoris was purified with Protein G column. The numbers showed the different eluted fractions. Figure S6. SDS-PAGE and lectin blo [file 12934_2020_1280_MOESM1_ESM.pptx]

## Slide 1
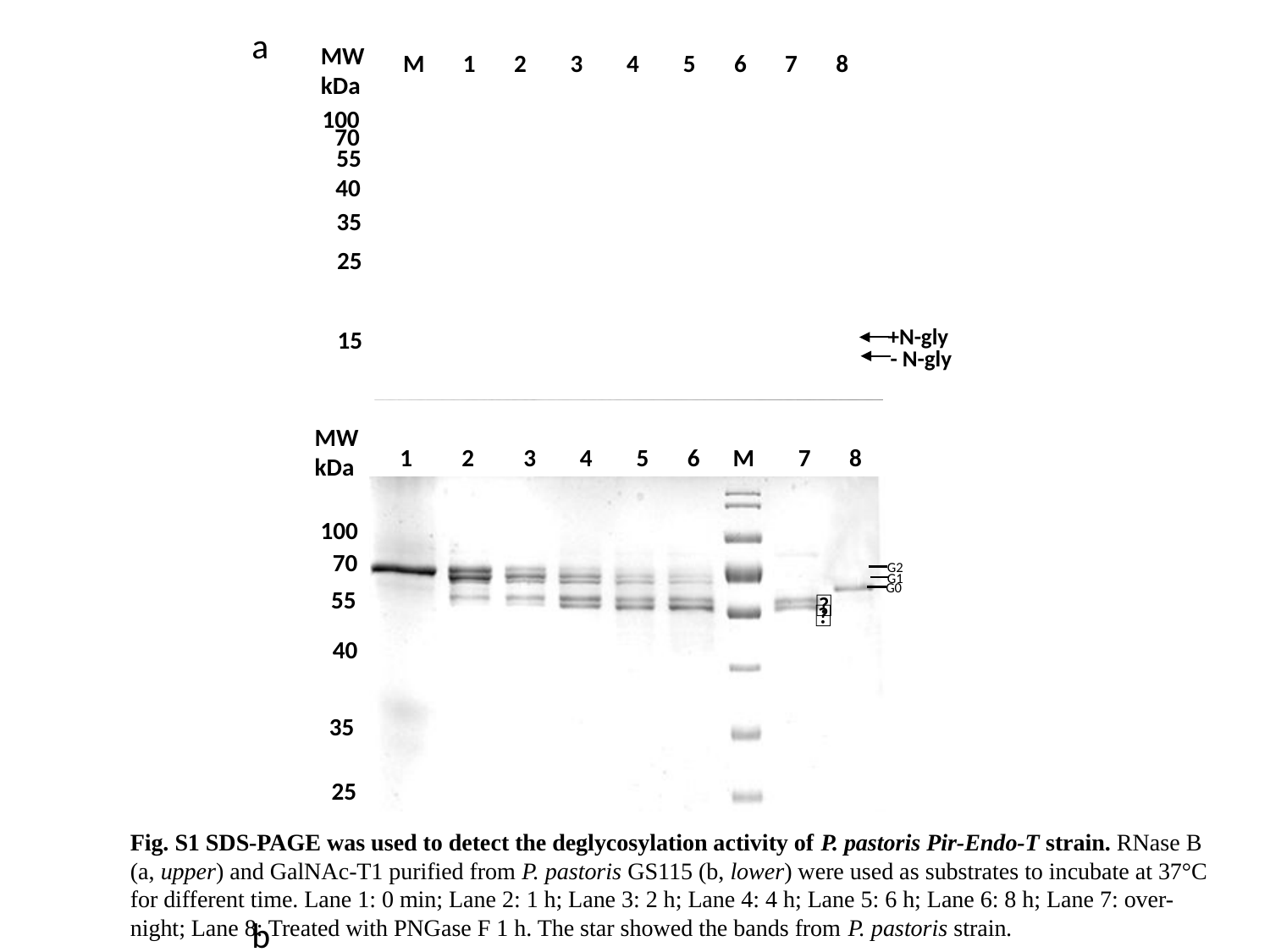

a
b
MW
kDa
 M 1 2 3 4 5 6 7 8
100
 70
 55
 40
 35
 25
 15
+N-gly
 - N-gly
MW
kDa
1 2 3 4 5 6 M 7 8
100
 70
55
 40
35
25
G2
G1
G0
﹡
﹡
Fig. S1 SDS-PAGE was used to detect the deglycosylation activity of P. pastoris Pir-Endo-T strain. RNase B (a, upper) and GalNAc-T1 purified from P. pastoris GS115 (b, lower) were used as substrates to incubate at 37°C for different time. Lane 1: 0 min; Lane 2: 1 h; Lane 3: 2 h; Lane 4: 4 h; Lane 5: 6 h; Lane 6: 8 h; Lane 7: over-night; Lane 8: Treated with PNGase F 1 h. The star showed the bands from P. pastoris strain.

## Slide 2
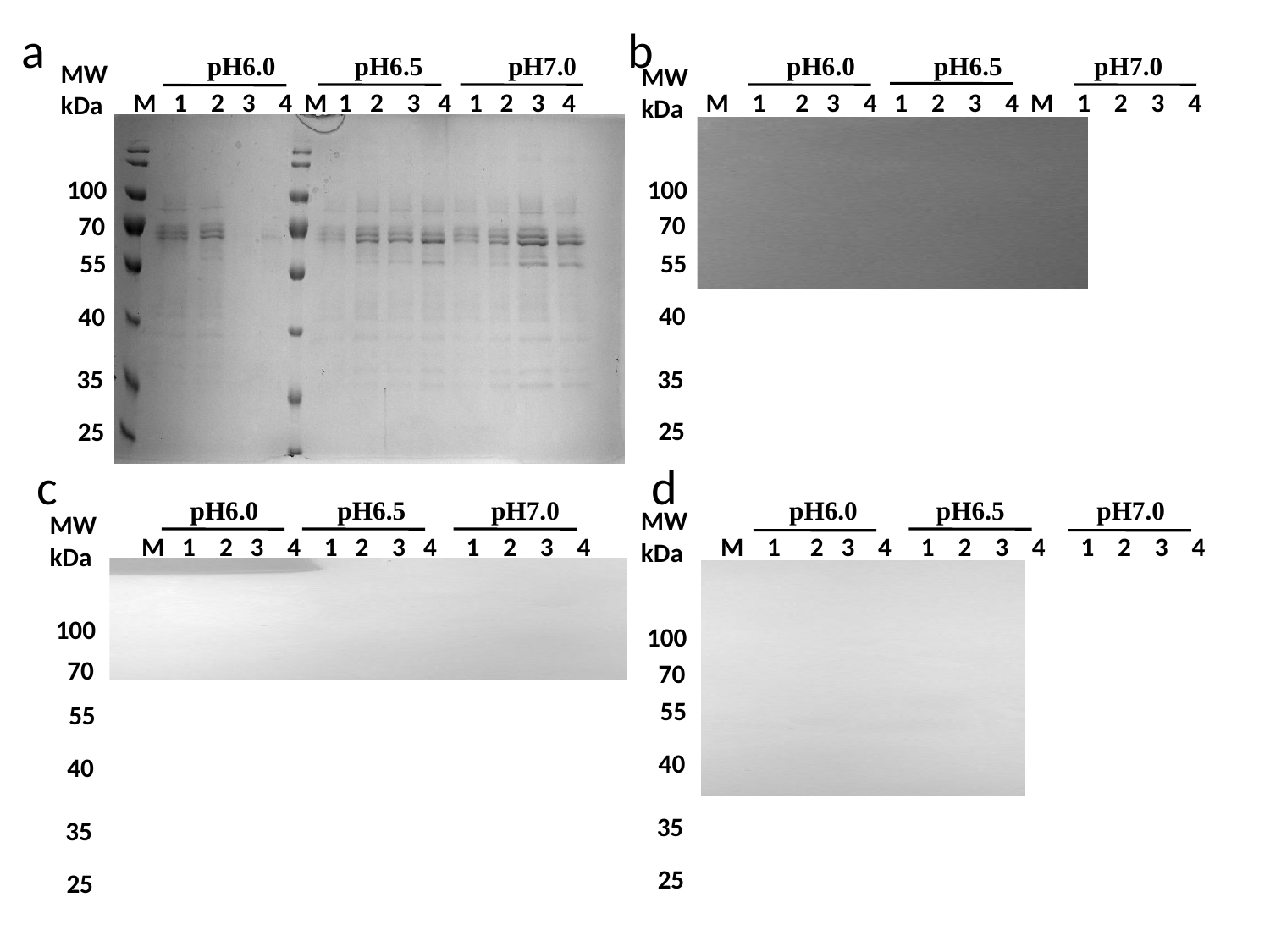

a b
pH6.0 pH6.5 pH7.0 pH6.0 pH6.5 pH7.0
MW
kDa
100
 70
55
 40
35
25
MW
kDa
100
 70
55
 40
35
25
M 1 2 3 4 M 1 2 3 4 1 2 3 4 M 1 2 3 4 1 2 3 4 M 1 2 3 4
c d
MW
kDa
100
 70
55
 40
35
25
pH6.0 pH6.5 pH7.0 pH6.0 pH6.5 pH7.0
M 1 2 3 4 1 2 3 4 1 2 3 4 M 1 2 3 4 1 2 3 4 1 2 3 4
MW
kDa
100
 70
55
 40
35
25

## Slide 3
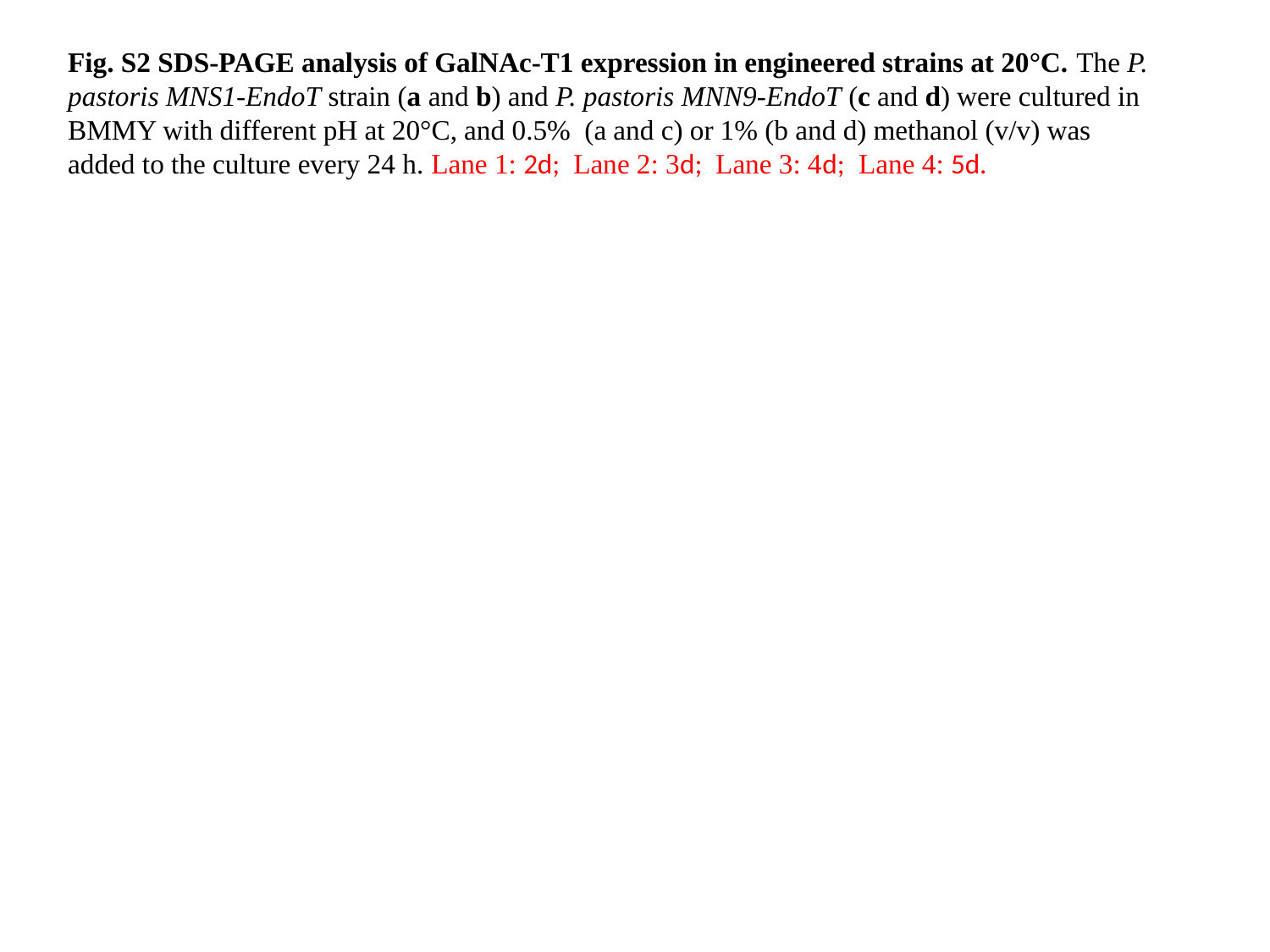

Fig. S2 SDS-PAGE analysis of GalNAc-T1 expression in engineered strains at 20°C. The P. pastoris MNS1-EndoT strain (a and b) and P. pastoris MNN9-EndoT (c and d) were cultured in BMMY with different pH at 20°C, and 0.5% (a and c) or 1% (b and d) methanol (v/v) was added to the culture every 24 h. Lane 1: 2d; Lane 2: 3d; Lane 3: 4d; Lane 4: 5d.

## Slide 4
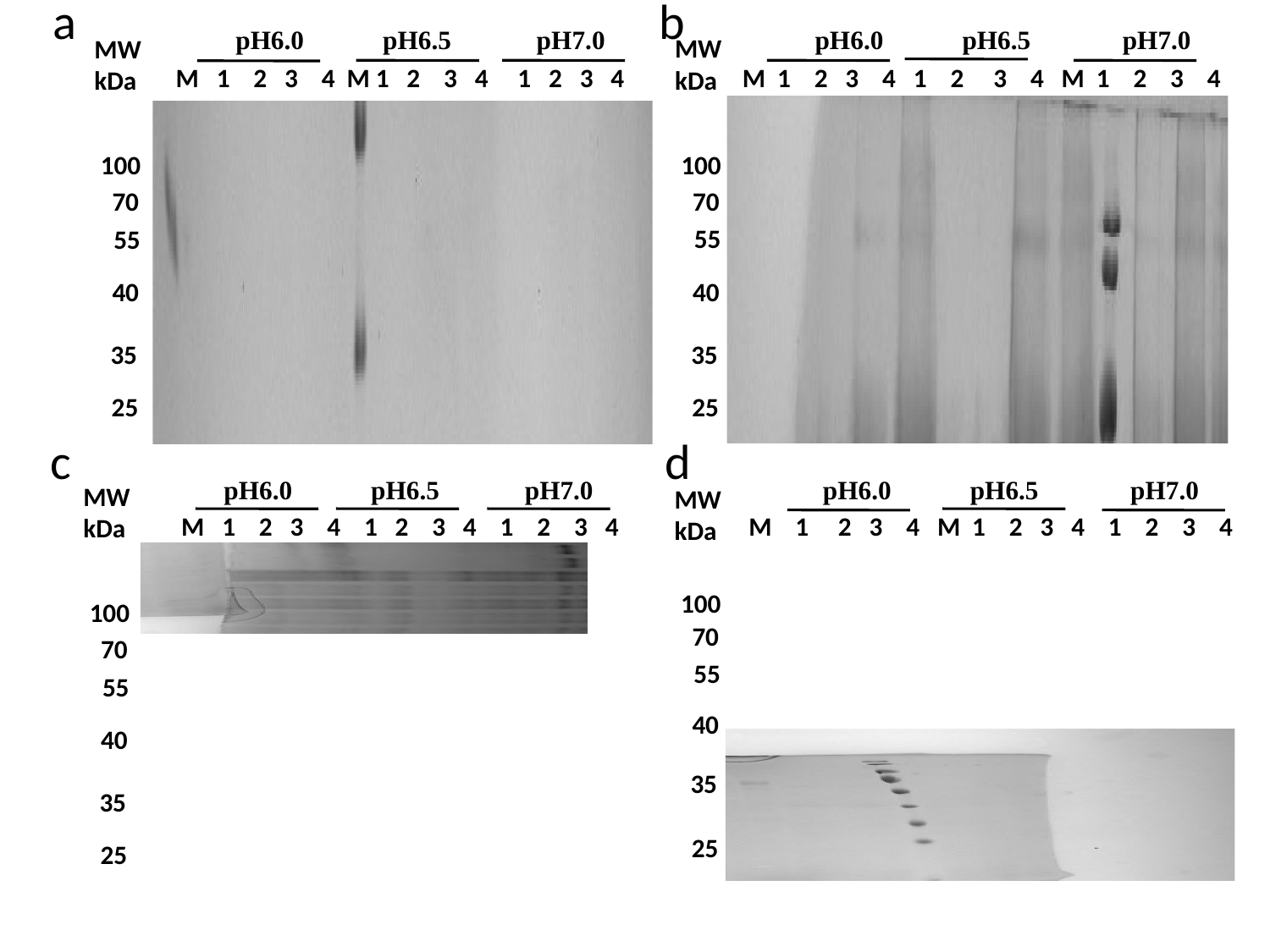

a b
pH6.0 pH6.5 pH7.0 pH6.0 pH6.5 pH7.0
MW
kDa
100
 70
55
 40
35
25
MW
kDa
100
 70
55
 40
35
25
M 1 2 3 4 M 1 2 3 4 1 2 3 4 M 1 2 3 4 1 2 3 4 M 1 2 3 4
c d
pH6.0 pH6.5 pH7.0 pH6.0 pH6.5 pH7.0
M 1 2 3 4 1 2 3 4 1 2 3 4 M 1 2 3 4 M 1 2 3 4 1 2 3 4
MW
kDa
100
 70
55
 40
35
25
MW
kDa
100
 70
55
 40
35
25

## Slide 5
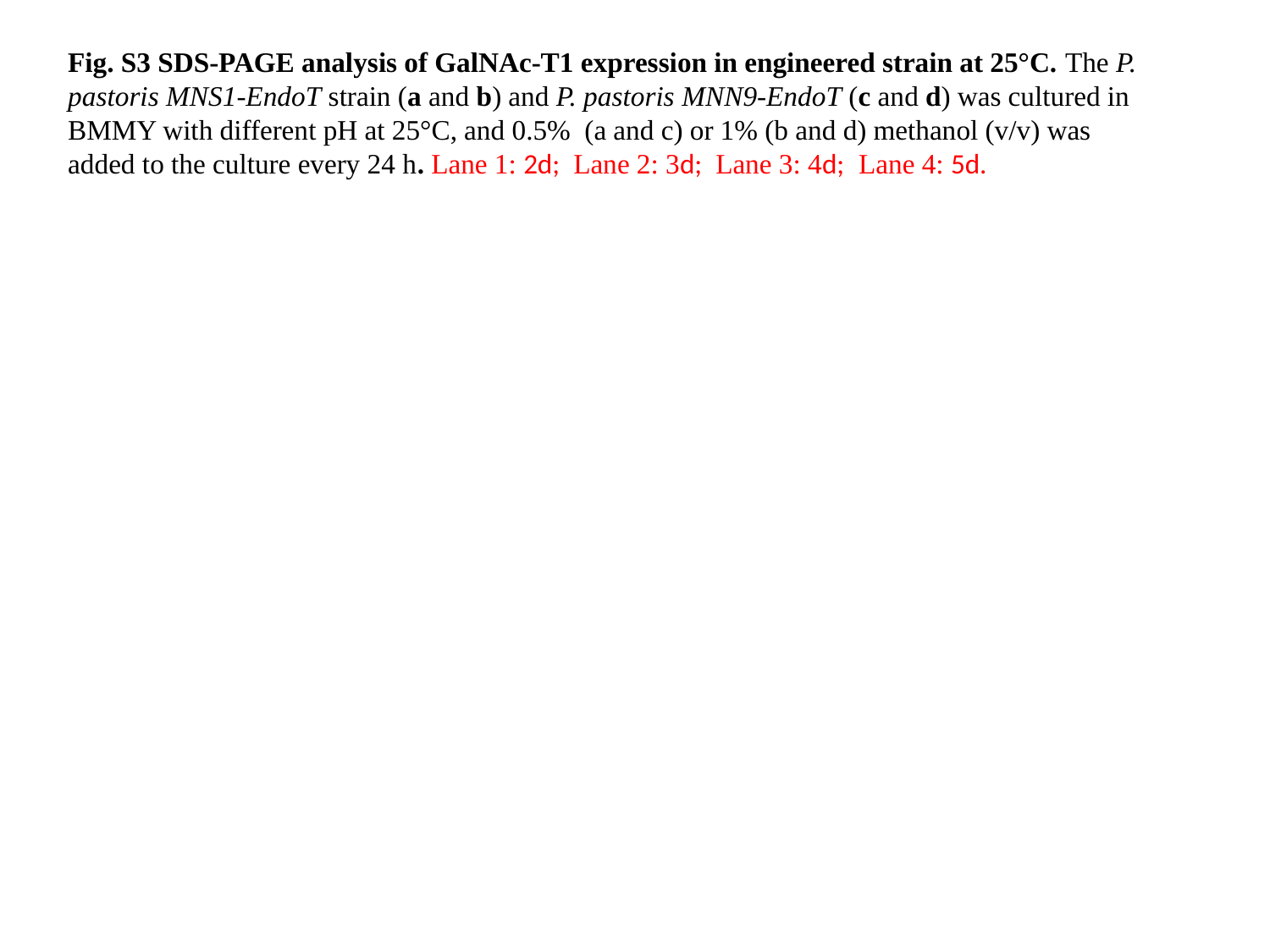

Fig. S3 SDS-PAGE analysis of GalNAc-T1 expression in engineered strain at 25°C. The P. pastoris MNS1-EndoT strain (a and b) and P. pastoris MNN9-EndoT (c and d) was cultured in BMMY with different pH at 25°C, and 0.5% (a and c) or 1% (b and d) methanol (v/v) was added to the culture every 24 h. Lane 1: 2d; Lane 2: 3d; Lane 3: 4d; Lane 4: 5d.

## Slide 6
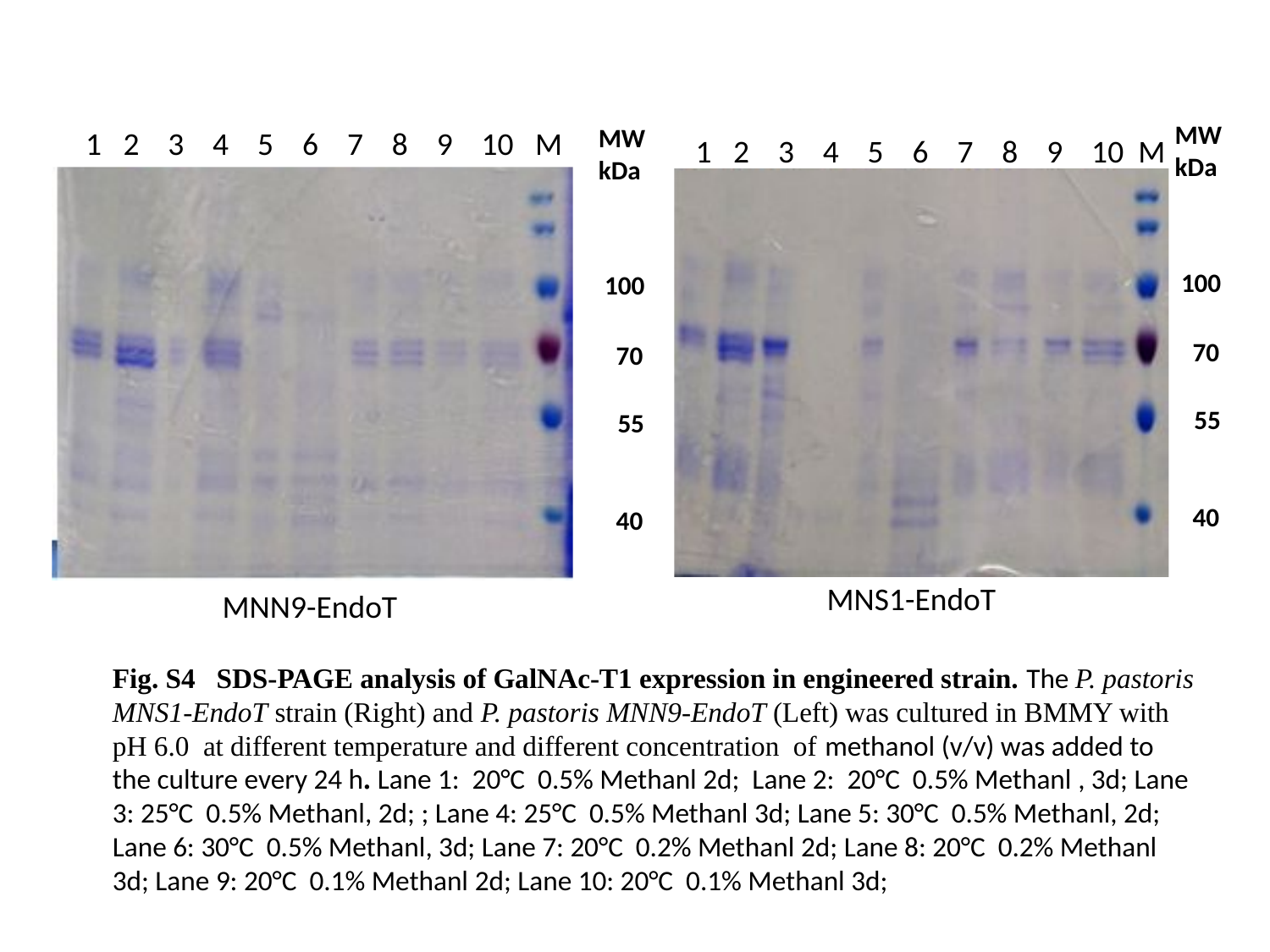

MW
kDa
100
 70
55
 40
MW
kDa
100
 70
55
 40
1 2 3 4 5 6 7 8 9 10 M
1 2 3 4 5 6 7 8 9 10 M
MNS1-EndoT
MNN9-EndoT
Fig. S4 SDS-PAGE analysis of GalNAc-T1 expression in engineered strain. The P. pastoris MNS1-EndoT strain (Right) and P. pastoris MNN9-EndoT (Left) was cultured in BMMY with pH 6.0 at different temperature and different concentration of methanol (v/v) was added to the culture every 24 h. Lane 1: 20°C 0.5% Methanl 2d; Lane 2: 20°C 0.5% Methanl , 3d; Lane 3: 25°C 0.5% Methanl, 2d; ; Lane 4: 25°C 0.5% Methanl 3d; Lane 5: 30°C 0.5% Methanl, 2d; Lane 6: 30°C 0.5% Methanl, 3d; Lane 7: 20°C 0.2% Methanl 2d; Lane 8: 20°C 0.2% Methanl 3d; Lane 9: 20°C 0.1% Methanl 2d; Lane 10: 20°C 0.1% Methanl 3d;

## Slide 7
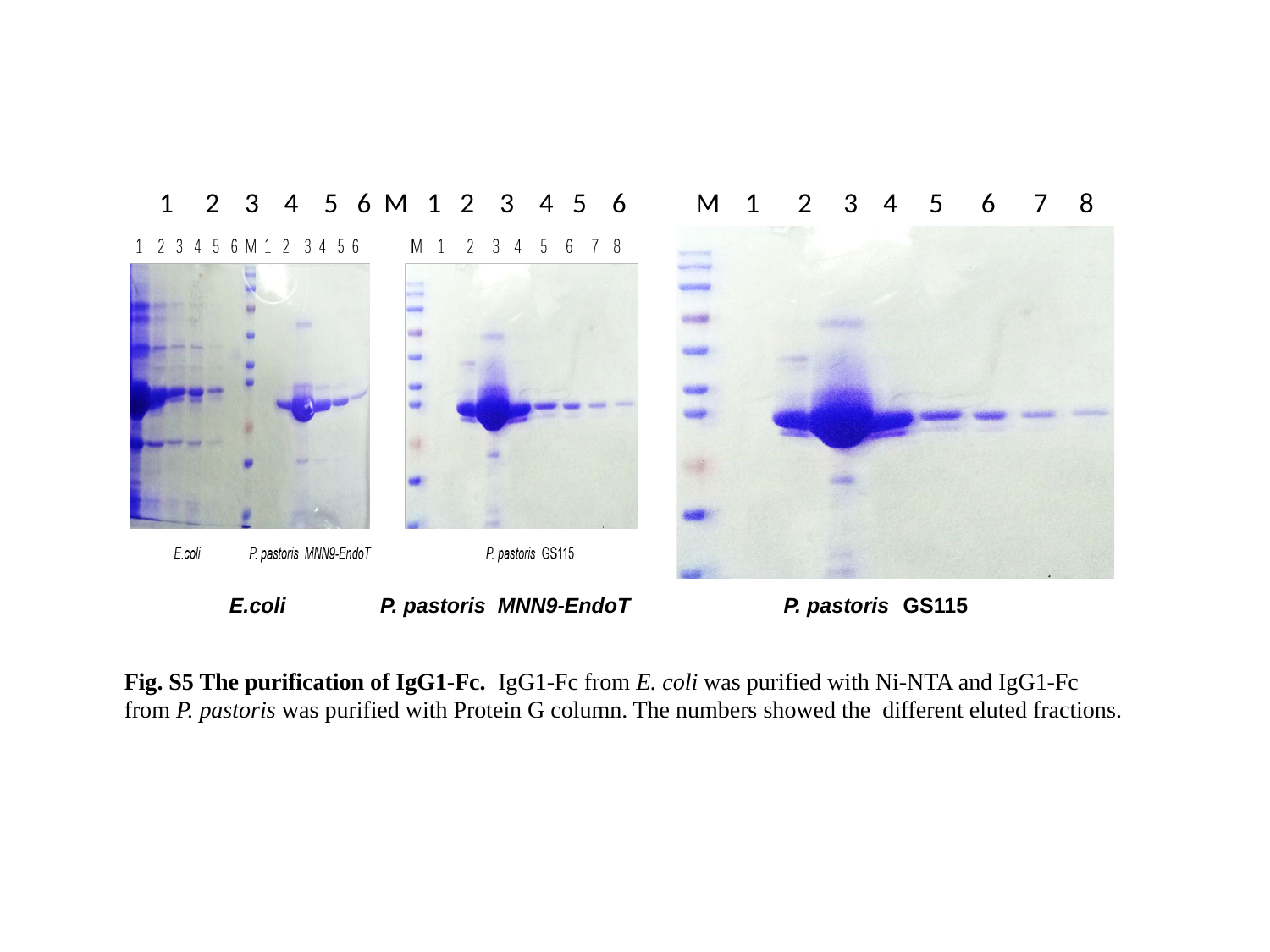

1 2 3 4 5 6 M 1 2 3 4 5 6 M 1 2 3 4 5 6 7 8
 E.coli P. pastoris MNN9-EndoT P. pastoris GS115
Fig. S5 The purification of IgG1-Fc. IgG1-Fc from E. coli was purified with Ni-NTA and IgG1-Fc from P. pastoris was purified with Protein G column. The numbers showed the different eluted fractions.

## Slide 8
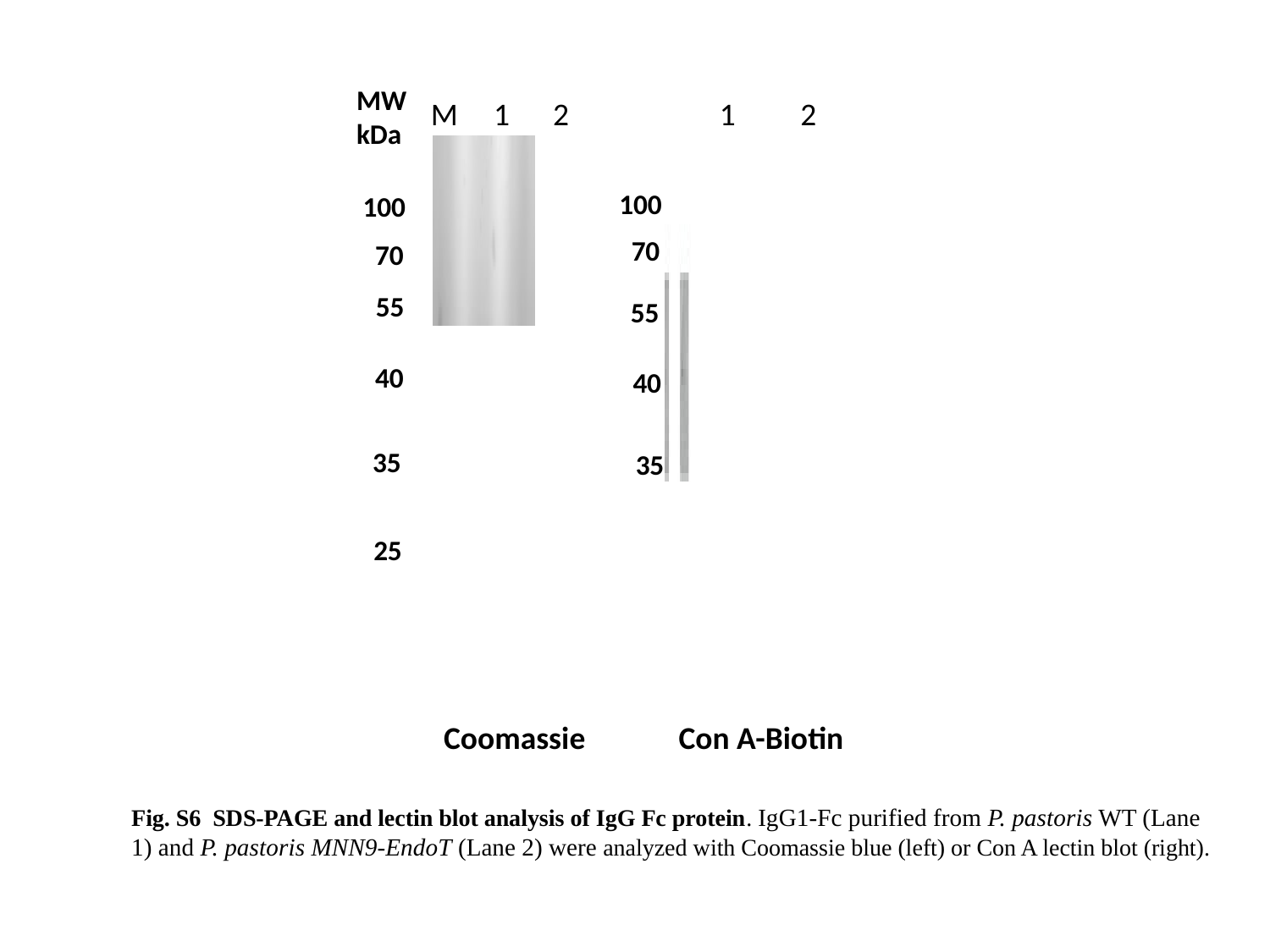

MW
kDa
100
 70
55
 40
35
25
M 1 2 1 2
Coomassie Con A-Biotin
100
 70
55
 40
35
Fig. S6 SDS-PAGE and lectin blot analysis of IgG Fc protein. IgG1-Fc purified from P. pastoris WT (Lane 1) and P. pastoris MNN9-EndoT (Lane 2) were analyzed with Coomassie blue (left) or Con A lectin blot (right).

## Slide 9
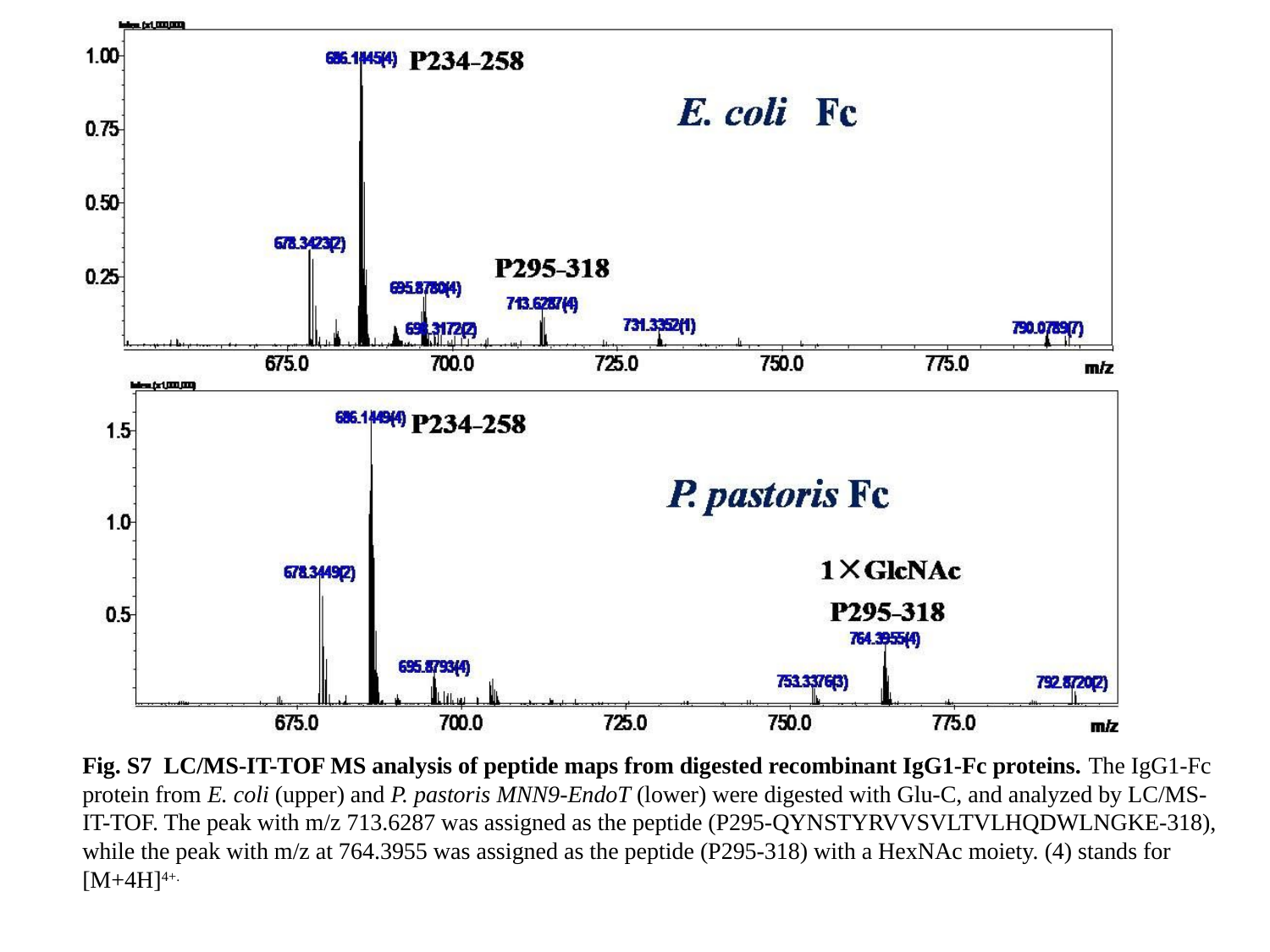

Fig. S7 LC/MS-IT-TOF MS analysis of peptide maps from digested recombinant IgG1-Fc proteins. The IgG1-Fc protein from E. coli (upper) and P. pastoris MNN9-EndoT (lower) were digested with Glu-C, and analyzed by LC/MS-IT-TOF. The peak with m/z 713.6287 was assigned as the peptide (P295-QYNSTYRVVSVLTVLHQDWLNGKE-318), while the peak with m/z at 764.3955 was assigned as the peptide (P295-318) with a HexNAc moiety. (4) stands for [M+4H]4+.
