# Supplementary material for: Homogeneous production and characterization of recombinant N-GlcNAc-protein in Pichia pastoris
Source: Microb Cell Fact. 2020 Jan 13;19:7. doi: 10.1186/s12934-020-1280-0 (PMC6956495; doi:10.1186/s12934-020-1280-0)
Supplement: Supplementary file 2 — Additional file 2: Table S1. The DNA and amino acid sequences used in this study. Table S2. Peptide map of recombinant IgG1-Fc domain digested with Glc-C. [file 12934_2020_1280_MOESM2_ESM.docx]

**Table S1. The DNA and amino acid sequences used in this study**

| **The name of gene** | **sequence** |
| --- | --- |
| **MNN9-TM** | GAATTCGCCACCATGTCACTTTCTCTTGTATCGTACCGCCTAAGAAAGAACCCGTGGGTTAACATTTTTCTACCTGTTTTGGCCATATTTCTAATATATATAATTTTTTTCCAGAGAGATCAATCTCTGTTGCTCGAG |
|  | MSLSLVSYRLRKNPWVNIFLPVLAIFLIYIIFFQRDQSLLLE |
| **MNS1-TM** | GAATTCGCCACCATGAAGAACTCTGTCGGTATTTCAATTGCAACCATTGTTGCTATCATAGCAGCTATATACTATGTGCCATGGTACGAACACTTTGAGAGACTCGAG |
|  | MKNSVGISIATIVAIIAAIYYVPWYEHFERLE |
| **Endo T** | CTCGAGGTTCCTGTCAAGGAGTTGCAATTGAGAGCCGAGCCAACTGACTTGCCAAGATTGATTGTTTACTTCCAGACTACTCACGACTCTTCCAACCGTCCAATCTCCATGTTGCCATTGATCACCGAGAAGGGTATTGCCTTGACCCACTTGATCGTCTGCTCCTTTCACATCAACCAAGGTGGAGTCGTCCACTTGAACGACTTCCCACCAGATGACCCTCACTTCTACACCTTGTGGAACGAGACCATCACCATGAAGCAAGCTGGAGTCAAGGTCATGGGTATGGTTGGAGGTGCCGCTCCAGGTTCTTTTAACACCCAGACCTTGGACTCTCCTGACTCCGCCACCTTCGAGCATTACTACGGTCAATTGAGAGATGCCATTGTTAACTTTCAATTGGAAGGAATGGATTTGGACGTCGAACAACCAATGTCCCAGCAGGGTATTGACAGATTGATCGCCCGTTTGAGAGCTGACTTCGGTCCAGATTTCCTTATTACCTTGGCTCCAGTTGCCTCCGCTTTGGAAGACTCCTCCAACTTGTCCGGTTTCTCCTACACTGCTTTGCAGCAGACTCAGGGTAACGACATCGACTGGTACAACACCCAGTTCTACTCTGGTTTCGGTTCCATGGCCGACACTTCTGACTACGACCGTATCGTCGCTAACGGATTCGCCCCAGCCAAGGTTGTTGCCGGTCAATTGACTACCCCAGAAGGTGCTGGATGGATTCCTACTTCTTCCTTGAACAACACTATCGTCTCCTTGGTCTCCGAATACGGTCAGATCGGAGGTGTTATGGGATGGGAGTACTTCAACTCCTTGCCTGGTGGTACTGCTGAACCATGGGAATGGGCCCAAATCGTTACCGAAATTTTACGTCCTGGTTTGGTCCCAGAGCTTAAGATCACTGAGGACGACGCCGCTAGATTGACTGGTGCCTACGAGGAGTCTGTTAAGGCTGCCGCCGCTGACAACAAATCTTTCGTCAAGAGACCATCTATTAACTATTACGCCATGGTTAACGCCGACTACAAGGACGATGACGATAAGTAAGCGGCCGC |
|  | LEVPVKELQLRAEPTDLPRLIVYFQTTHDSSNRPISMLPLITEKGIALTHLIVCSFHINQGGVVHLNDFPPDDPHFYTLWNETITMKQAGVKVMGMVGGAAPGSFNTQTLDSPDSATFEHYYGQLRDAIVNFQLEGMDLDVEQPMSQQGIDRLIARLRADFGPDFLITLAPVASALEDSSNLSGFSYTALQQTQGNDIDWYNTQFYSGFGSMADTSDYDRIVANGFAPAKVVAGQLTTPEGAGWIPTSSLNNTIVSLVSEYGQIGGVMGWEYFNSLPGGTAEPWEWAQIVTEILRPGLVPELKITEDDAARLTGAYEESVKAAAADNKSFVKRPSINYYAMVNADYKDDDDK* |
| **IgG1 Fc region** | GGATCCGAACCCAAGTCCTGCGACAAGACCCACACCTGTCCCCCTTGTCCTGCCCCTGAACTGCTGGGCGGACCCAGCGTGTTCCTGTTCCCCCCAAAGCCCAAGGATACCCTGATGATCTCCCGGACCCCCGAAGTGACCTGCGTGGTGGTGGATGTGTCCCACGAGGACCCTGAAGTGAAGTTCAATTGGTACGTGGACGGCGTGGAAGTGCACAACGCCAAGACCAAGCCTAGAGAGGAACAGTACAACTCCACCTACCGGGTGGTGTCCGTGCTGACCGTGCTGCACCAGGATTGGCTGAACGGCAAAGAGTACAAGTGCAAGGTGTCCAACAAGGCCCTGCCTGCCCCCATCGAAAAGACCATCTCCAAGGCCAAGGGCCAGCCCCGGGAACCCCAGGTGTACACACTGCCCCCTAGCAGGGACGAGCTGACCAAGAACCAGGTGTCCCTGACATGCCTCGTGAAAGGCTTCTACCCCTCCGATATCGCCGTGGAATGGGAGTCCAACGGCCAGCCTGAGAACAACTACAAGACCACCCCCCCTGTGCTGGACTCCGACGGCTCATTCTTCCTGTACAGCAAGCTGACAGTGGACAAGTCCCGGTGGCAGCAGGGCAACGTGTTCTCCTGCTCCGTGATGCACGAGGCCCTGCACAACCACTATACCCAGAAGTCCCTGTCCCTGAGCCCCGGCAAGTGACTCGAG |
|  | GSEPKSCDKTHTCPPCPAPELLGGPSVFLFPPKPKDTLMISRTPEVTCVVVDVSHEDPEVKFNWYVDGVEVHNAKTKPREEQYNSTYRVVSVLTVLHQDWLNGKEYKCKVSNKALPAPIEKTISKAKGQPREPQVYTLPPSRDELTKNQVSLTCLVKGFYPSDIAVEWESNGQPENNYKTTPPVLDSDGSFFLYSKLTVDKSRWQQGNVFSCSVMHEALHNHYTQKSLSLSPGK |
| **GALNT1** | ggacttcctgctggagatgttctagagccagtacaaaagcctcatgaaggtcctggagaaatggggaaaccagtcgtcattcctaaagaggatcaagaaaagatgaaagagatgtttaaaatcaatcagttcaatttaatggcaagtgagatgattgcactcaacagatctttaccagatgttaggttagaagggtgtaaaacaaaggtgtatccagataatcttcctacaacaagtgtggtgattgttttccacaatgaggcttggagcacacttctgcgaactgtccatagtgtcattaatcgctcaccaagacacatgatagaagaaattgttctagtagatgatgccagtgaaagagactttttgaaaaggcctttagagagttatgtgaaaaaactaaaagtaccagttcatgtaattcgaatggaacaacgttctggattgatcagagctagattaaaaggagctgctgtgtctaaaggccaagtgatcaccttcctggatgcccattgtgagtgtacagtgggatggctggagcctctcttggccaggatcaaacatgacaggagaacagtggtgtgtcccatcatcgatgtgatcagtgatgatacttttgagtacatggcaggctctgatatgacctatggtgggttcaactggaagctcaattttcgctggtatcctgttccccaaagagaaatggacagaaggaaaggtgatcggactcttcctgtcaggacacctaccatggcaggaggccttttttcaatagacagagattactttcaggaaattggaacatatgatgctggaatggatatttggggaggagaaaacctagaaatttcctttaggatttggcagtgtggaggaactttggaaattgttacatgctcacatgttggacatgtgtttcggaaagctacaccttacacgtttccaggaggcacagggcagattatcaataaaaataacagacgacttgcagaagtgtggatggatgaattcaagaatttcttctatataatttctccaggtgttacaaaggtagattatggagatatatcgtcaagagttggtctaagacacaaactacaatgcaaacctttttcctggtacctagagaatatatatcctgattctcaaattccacgtcactatttctcattgggagagatacgaaatgtggaaacgaatcagtgtctagataacatggctagaaaagagaatgaaaaagttggaatttttaattgccatggtatggggggtaatcaggttttctcttatactgccaacaaagaaattagaacagatgacctttgcttggatgtttccaaacttaatggcccagttacaatgctcaaatgccaccacctaaaaggcaaccaactctgggagtatgacccagtgaaattaaccctgcagcatgtgaacagtaatcagtgcctggataaagccacagaagaggatagccaggtgcccagcattagagactgcaatggaagtcggtcccagcagtggcttcttcgaaacgtcaccctgccagaaatattcCATCATCATCATCATCACTAG |
|  | GLPAGDVLEPVQKPHEGPGEMGKPVVIPKEDQEKMKEMFKINQFNLMASEMIALNRSLPDVRLEGCKTKVYPDNLPTTSVVIVFHNEAWSTLLRTVHSVINRSPRHMIEEIVLVDDASERDFLKRPLESYVKKLKVPVHVIRMEQRSGLIRARLKGAAVSKGQVITFLDAHCECTVGWLEPLLARIKHDRRTVVCPIIDVISDDTFEYMAGSDMTYGGFNWKLNFRWYPVPQREMDRRKGDRTLPVRTPTMAGGLFSIDRDYFQEIGTYDAGMDIWGGENLEISFRIWQCGGTLEIVTCSHVGHVFRKATPYTFPGGTGQIINKNNRRLAEVWMDEFKNFFYIISPGVTKVDYGDISSRVGLRHKLQCKPFSWYLENIYPDSQIPRHYFSLGEIRNVETNQCLDNMARKENEKVGIFNCHGMGGNQVFSYTANKEIRTDDLCLDVSKLNGPVTMLKCHHLKGNQLWEYDPVKLTLQHVNSNQCLDKATEEDSQVPSIRDCNGSRSQQWLLRNVTLPEIFHHHHHH* |

**TableS2. Peptide map of recombinant IgG1-Fc domain digested with Glc-C**

| **Peptide*** | **Sequence** | **Peptide mass [Da]** | | |
| --- | --- | --- | --- | --- |
| P217-233 | PKSCDKTHTCPPCPAPE | 1811.076 | | |
| P234-258 | LLGGPSVFLFPPKPKDTLMISRTPE | 2741.284 | | |
| P259-269 | VTCVVVDVSHE | 1186.346 | | |
| P273-283 | VKFNWYVDGVE | 1355.513 | | |
| P284-80 | VHNAKTKPRE | 1179.345 | | |
| P295-318 | QYNSTYRVVSVLTVLHQDWLNGKE | 2850.183 | | |
| P319-333 | YKCKVSNKALPAPIE | 1660.992 | | |
| P334-345 | KTISKAKGQPRE | 1342.561 | | |
| P346-357 | PQVYTLPPSRDE | 1401.539 | | |
| P358-380 | LTKNQVSLTCLVKGFYPSDIAVE | 2525.943 | | |
| P389-430 | NNYKTTPPVLDSDGSFFLYSKLTVDKSRWQQGNVFSCSVMHE | 4827.377 | | |
| P431-447 | ALHNHYTQKSLSLSPGK | 1881.121 | | |
|  |  |  |  |  |

* The number of amino acid from full length IgG1 protein.
